# Identification of *FEZ2* as a potential oncogene in pancreatic ductal adenocarcinoma

**DOI:** 10.7717/peerj.12736

**Published:** 2022-01-05

**Authors:** Chaozhi Yang, Xuebing Wang, Chenjie Qiu, Ziruo Zheng, Kai Lin, Min Tu, Kai Zhang, Kuirong Jiang, Wentao Gao

**Affiliations:** 1 Pancreas Center, The First Affiliated Hospital of Nanjing Medical University, Nanjing, Jiangsu Province, China; 2 Pancreas Institute, Nanjing Medical University, Nanjing, Jiangsu Province, China

**Keywords:** Pancreatic ductal adenocarcinoma, FEZ2, Prognosis, Chemotherapy resistance, Wnt

## Abstract

Pancreatic ductal adenocarcinoma (PDAC) is one of the common malignant tumors with high lethal rate and poor prognosis. Dysregulation of many genes have been reported to be involved in the occurrence and development of PDAC. However, as a highly conserved gene in eukaryotes, the role of Fasciculation and Elongation protein Zeta 2 (FEZ2) in pancreatic cancer progression is not clear. In this study, we identified the oncogenic effect of FEZ2 on PDAC. By mining of The Cancer Genome Atlas (TCGA) database, we found that FEZ2 was upregulated in PDAC tissues and FEZ2 expression was negatively regulated by its methylation. Moreover, high expression and low methylation of FEZ2 correlated with poor prognosis in PDAC patients. Besides, we found that FEZ2 could promote PDAC cells proliferation, migration and 5-FU resistance *in vitro*. Furthermore, Gene pathway enrichment analysis demonstrated a positive correlation between Wnt signaling activation and FEZ2 expression in PDAC patients. Western blot showed that FEZ2 knockdown significantly suppressed β-catenin expression. Collectively, our finding revealed that FEZ2 functioned as a potential oncogene on PDAC progression and migration, and the expression of FEZ2 had guidance value for the treatment and chemotherapy program of PDAC patients.

## Introduction

Pancreatic ductal adenocarcinoma (PDAC) is one of the most aggressive gastrointestinal malignancies with a five-year survival rate less than 5% and median survival time around 6 months (Ilic & Ilic, 2016). The high lethal rate and poor prognosis of PDAC is due to many factors, including the difficulty of early diagnosis because of the lesion site and different response to chemotherapy caused by the complexity of genetic background and tumor microenvironment (Gnanamony & Gondi, 2017; Singhi et al., 2019). Currently, chemotherapy is one of the effective adjuvant therapies for PDAC, while 5-Fluorouracil (5-FU) is one of the first-line chemotherapeutic agents (Tamburrino et al., 2013). However, many patients have limited response to this therapy due to 5-FU resistance (Katz et al., 2017). Therefore, it is urgent to understand the molecular and cellular mechanisms of PDAC progression and 5-FU resistance.

In order to find genes which promoted the progression of PDAC, we firstly searched dysregulated genes in patients by data mining. Interestingly, we found a group of conserved genes family, the Fasciculation and Elongation Zeta (FEZ) protein family, is dysregulated in many cancer types. Usually the highly conserved genes are thought to be have essential functions during the biological process (Bryan et al., 2020). Also, many studies revealed that dysregulation of conserved genes play important role in cancer progression (Colak et al., 2010; Khalil et al., 2018). This led us to focusing on the role of FEZ family in PDAC.

The FEZ protein family was first described in 1997 by Bloom & Horvitz (1997) as a highly conserved family with *C. elegans* unc-76 gene. This family has two members in human: FEZ1 and FEZ2. Previous study showed that FEZ1 could rescue the defects in axon-axon interaction during brain development caused by unc-76 mutation, this suggested that FEZ1 shared evolutionary conserved functions from *C. elegans* to Human (Fujita et al., 2004; Gindhart et al., 2003). However, little is known about the function of FEZ2. The protein-protein interactions (PPI) showed FEZ2 could interacted with more proteins than FEZ1 and unc-76, indicating that FEZ2 might achieve functional evolution through specific protein interactions (Alborghetti, Furlan & Kobarg, 2011).

In this study, we first explored the expression profile of FEZ family in many cancer types. Both FEZ1 and FEZ2 showed dysregulation in many cancer types, but only few types of cancer showed an upregulation of FEZ2, which including PDAC. Since there is no study reported that FEZ family play an oncogenic role in any type of cancer previously, this led us to examine the function of FEZ2 in PDAC. Interestingly, inhibition of FEZ2 expression results in a reduction of PDAC cells proliferation. Then we made more analysis to illustrate the function of FEZ2 in PDAC.

We revealed the aberrant expression of FEZ2 in PDAC tissues and investigated the association between FEZ2 expression and FEZ2 methylation based on The Cancer Genome Atlas (TCGA) database. After we examined the prognostic role of FEZ2 expression and its methylation. In addition, we analyzed the correlation between FEZ2 expression and chemotherapy resistance including the first-line chemotherapeutic agents Gemcitabine and 5-FU. Moreover, we investigated the correlation between FEZ2 expression and immune cell infiltration by using TIMER database. Analysis about miRNA regulation of FEZ2 was also included into this study. Furthermore, we performed the Gene set enrichment analysis (GSEA) to identify the potential functional mechanism of FEZ2 in PDAC, and validated the pathway changing by western blot. Finally, we validated the function of FEZ2 on tumor cell proliferation, migration and 5-FU resistance by using PDAC cell lines and clinical samples.

## Materials and Methods

### Data mining from public databases

FEZ1 and FEZ2 expression in different cancers was obtained from Oncomine database (https://www.oncomine.org/) (Rhodes et al., 2004). To investigate FEZ2 expression in pancreatic cancer tissues and normal pancreas tissues, we downloaded the expression data from TCGA database (https://www.cancer.gov/tcga) and GTEx database *via* UCSC Xena website (https://xenabrowser.net/) (Lonsdale et al., 2013). Copy number and methylation of FEZ2 were also obtained from TCGA data *via* UCSC Xena website. FEZ2 expression in Logsdon Pancreas dataset and Badea Pancreas dataset were obtained from Oncomine database.

### Prognostic analysis of FEZ2 expression and methylation

We assessed the prognostic analysis of FEZ2 expression using DrBioRight (Li et al., 2021), a AI-driven analytics platform which could explore cancer omics data online. The patients were divided into two FEZ2 high expression group and FEZ2 low expression based on the median of FEZ2 expression. To examine the prognostic significance of FEZ2, Cox regression and log-rank test were performed. MethSurv (https://biit.cs.ut.ee/methsurv/), an online tool which could perform prognostic analysis using DNA methylation data (Modhukur et al., 2018), was used to investigate the prognostic role of FEZ2 methylation. The methylation landscape of FEZ2 promoter region was also obtained from MethSurv website.

### Chemotherapy resistance analysis

The expression of FEZ2 on PDAC cell lines were obtained from CCLE (Cancer Cell Line Encyclopedia) database (https://portals.broadinstitute.org/), the IC50 (half maximal inhibitory concentration) of Gemcitabine and 5-FU in different PDAC cell lines were obtained from GDSC (Genomics of Drug Sensitivity in Cancer) database (https://www.cancerrxgene.org/). Correlation analysis was performed between FEZ2 expression and IC50 of Gemcitabine and 5-FU.

### Immune cell infiltration analysis

We used TIMER database (https://cistrome.shinyapps.io/timer/) to predict the association between immune infiltration and FEZ2 expression (Li et al., 2017). The correlation between FEZ2 and six kinds of immune cells including B cell, CD8+ T cell, CD4+ T cell, macrophage, neutrophil and dendritic cell in PDAC were performed *via* TIMER. Then, we explored the association of FEZ2 with tumor purity and cancer associated fibroblast. In addition, we investigated the relationships between FEZ2 and immunotherapy targets including PD-1, PD-L1 and CTLA4.

### miRNA regulation of FEZ2

Online tools including TargetScan, miRWalk, miRDB and starBase were used to predict FEZ2 targeted miRNAs. Results from different tools were overlapped together. Then, the expression of miRNAs in PDAC tissues were obtained from TCGA data. In addition, the survival analysis of miR-433 was performed *via* UALCAN tools (http://ualcan.path.uab.edu/) (Chandrashekar et al., 2017).

### Functional enrichment analysis

Patients from TCGA database were divided into high FEZ2 group and low FEZ2 group. Differently expressed genes (DEGs) with |logFC| > 1.326 combined with *P* < 0.05 were identified as significant. To predict the potential functions of these genes, we performed functional enrichment analysis of DEGs to determine significantly enriched Gene Ontology (GO) terms and Kyoto Encyclopedia of Genes and Genomes (KEGG) pathway. The functional enrichment analysis was performed using cluster Profiler package on R software (Yu et al., 2012).

### Clinical samples

PDAC tissues (*n* = 50) and adjacent normal tissues (*n* = 50) were obtained from patients who had undergone surgical resection at Pancreas Center, The First Affiliated Hospital with Nanjing Medical University, Nanjing, China. PDAC tissues of patients who received 5-FU chemotherapy (*n* = 80) were also obtained from patients who had undergone surgical resection at Pancreas Center, The First Affiliated Hospital with Nanjing Medical University, Nanjing, China. Samples were collected after approved by the Ethics Committee of the First Affiliated Hospital of Nanjing Medical University (approval number 2018-SR-344) and written informed consent from the patients.

### Human Pancreatic cancer cell lines and culture

The human PC cell lines (BXPC-3, CFPAC, MIAPACA and PANC-1) and a normal human pancreatic ductal cell line (HPNE) that we used in this paper were obtained from ATCC. Conditions including Dulbecco’s modified Eagle’s medium (DMEM) (Wisent, Quebec, Canada) contained with 10% fetal bovine serum (Wisent, Quebec, Canada) at 37 °C in a 5% CO2 in humidified air.

### RNA extraction and RT-PCR

Total tissues or cells RNAs were extracted by using TRIzol reagent (Thermo Fisher Scientific). Prime Script RT Master Mix (Takara) was used for RNA reverse-transcription according to the manufacturer’s instructions. Quantitative PCR (Thunderbird SYBR qPCR mix, TOYOBO) were carried out in triplicate for the target genes. The levels of miRNAs were measured by qRT-PCR using miDETECT A Track™ miRNA qRT-PCR Kit (RiboBio, Guangzhou, China) The primers for miR-433-3p and U6 small nuclear RNA were purchased from RiboBio Company (Guangzhou, China). The sequences are covered by a patent. Target genes expression levels were determined using the method of delta-delta Ct (cycle threshold) and normalized with GAPDH or U6. Primer sets used for PCR: FEZ2-F ACGAGATTTGGAATGCCCTG and FEZ2-R CAAGGTCCTAGTATGCGATGAC; GAPDH-F GGAGCGAGATCCCTCCAAAAT and GAPDH-R GGCTGTTGTCATACTTCTCATGG; 18s rRNA-F AGCCACCCGAGATTGAGCA and 18s rRNA-R TAGTAGCGACGGGCGGTGTG. hsa-miR-433-3p mimics (Gene Pharma, Shanghai, China) 5′ to 3′ AUCAUGAUGGGCUCCUCGGU ACCGAGGAGCCCAUCAUGAUUU Negative control sense 5′-UUCUCCGAACGUGUCACGUTT-3′ antisense 5′-ACGUGACACGUUCGGAGAATT-3′.

### RNA interference

To silence FEZ2, two types of cancer cell lines CFPAC and PANC-1 were transfected with 50 nM siRNA (GenePharma, China) by using Lipofectamine 3000 (Thermo Fisher Scientific). After transfection 48 h, total cell RNA was extracted to validated FEZ2 expression by RT-PCR. Sequence of siRNA include: si-FEZ2#1 sense GAGCUUGUUAACUGAUUAUTT and antisense AUAAUCAGUUAAGCUCTT; si-FEZ2#2 sense GGACCCAGAAGAUGAUGAATT and antisense UUCAUCAUCUUCUGGGUCCTT.

### Western blot

Lysates of whole cell were extracted from PANC-1 and CFPAC cells. Fifty micrograms protein of each samples was separated by 10% SDS/PAGE gels, then transferred to nitrocellulose membranes by wet transfer. After blocked with 5% BSA in TBST (10 mM Tris, pH 8.0, 150 mM NaCl, 0.5% Tween 20) for 20 min, the membranes were washed twice with TBST and incubated with primary antibodies at 4 °C for 14 h. Membranes were washed three times with TBST for 10 min and incubated with secondary antibodies for 1 h at room temperature. After that, membrane were washed three times with TBST for 10 min. Finally, membrane was developed with the ECL (Amersham Biosciences) according to the manufacturer’s protocols. The primary antibodies we used for: anti-FEZ2 (sc-390111, 1:1,000, santa cruz), anti-GAPDH (A10868, 1:1,000, Abclonal), anti-β-actin (66009-1-1g, 1:1,000, proteintech), ani-β-catenin(SP328, 1:10,000, abcam).

### Drug sensitivity assay

PANC-1 and CFPAC cells were seeded into 96 well-plate at 1 × 10^4^ cells per well, and treated with 5-FU for 48 h. Cell viability was analyzed by CCK-8 assay (Beyotime Biotechnology). The assay was performed triplicate. Drug sensitivity was determined by IC50 (half maximal inhibitory concentration).

### Statistical analysis

Quantitative data were presented as the mean values ± S.D. with more than three independent experiments. Statistical differences between groups were calculated using Student’s t-test, one-way ANOVA, two-way ANOVA and Wilcoxon test on R software. The association between different factors was performed by Spearman’s correlation analysis. Chi-squared (χ^2^) test was used to investigate the association between FEZ2 expression and clinicopathological parameters. Kaplan–Meier analysis and log-rank test were used to examine the relationships between the survival rates and the expression of FEZ2 in PDAC patients. Cox proportional hazard regression model was used for univariate survival analysis. All statistical tests were two-tailed and *P* < 0.05 was considered as significant. Statistical significance was showed as *P < 0.05, **P < 0.01, ***P < 0.001.

## Results

### Expression of FEZ2 in pancreatic cancer and its correlation with copy number and methylation

**Figure 1:**
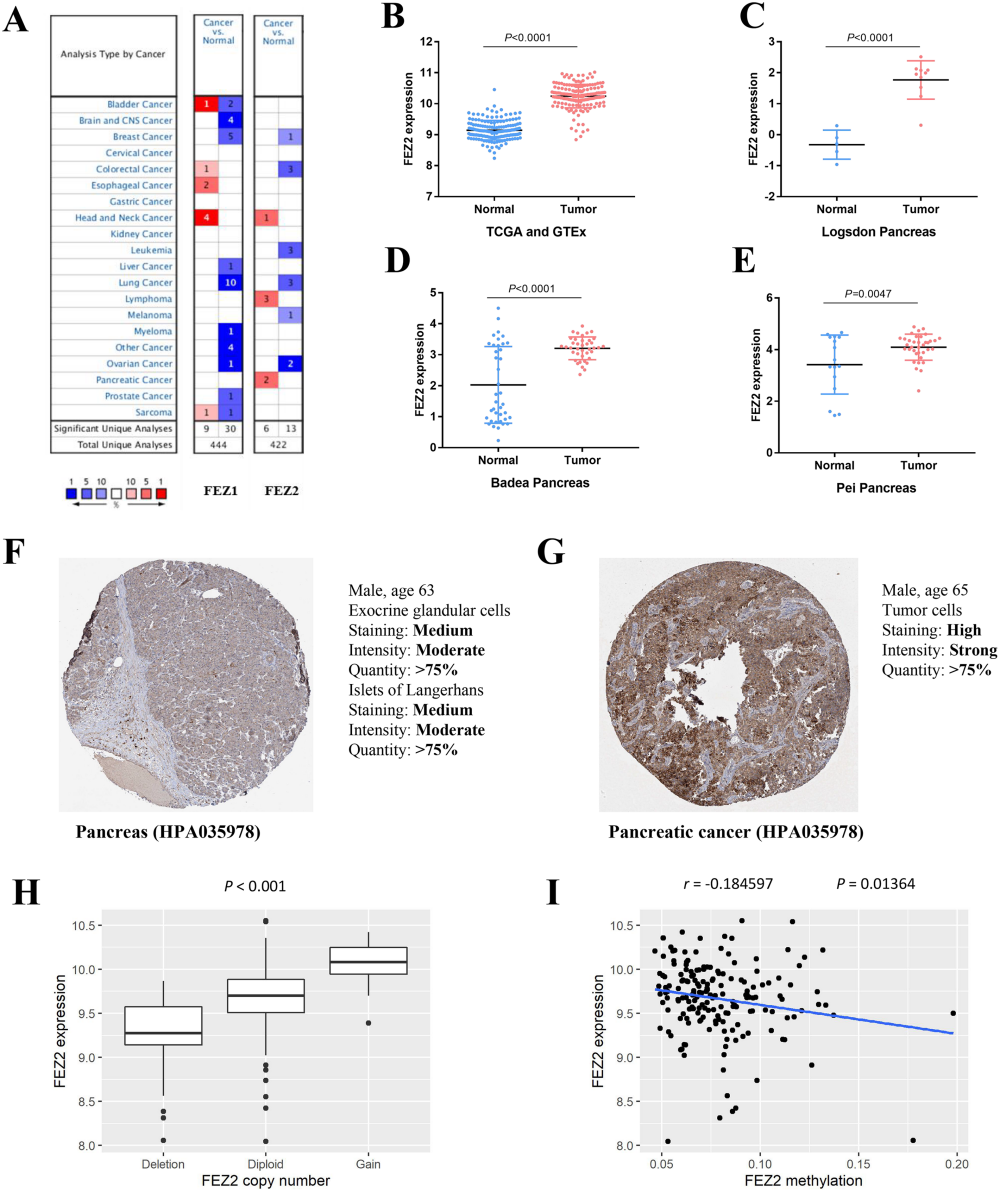
The expression of FEZ2 in PDAC and its correlation with copy number and methylation. (A) Expression profiles of FEZ1 and FEZ2 in different types of cancer. FEZ2 mRNA was highly expressed in PDAC tissues in TCGA and GTEx dataset. The number in each square indicated how many unique analyses showed significant results. (B), Logsdon Pancreas dataset (C), Badea Pancreas dataset (D) and Pei Pancreas dataset (E). (F, G) Immunohistochemistry from The Human Protein Atlas (THPA) showed FEZ2 was highly expressed in tumor cells. (H) FEZ2 mRNA expression was positively correlated with its copy number. (I) FEZ2 mRNA expression was negative correlated with FEZ2 DNA methylation.

To investigate the expression pattern of FEZ family in different cancer types, we searched Oncomine online website and found FEZ1 and FEZ2 were both upregulated in different cancer types (Fig. 1A). However, only FEZ2 was upregulated in pancreatic cancer. To further validate the expression pattern of FEZ2 in pancreatic cancer, we compared 178 PDAC tissues from TCGA database with 165 normal pancreas tissues from GTEx database (Fig. 1B). The results showed that FEZ2 was significantly upregulated in PDAC tissues. Logsdon Pancreas dataset (Fig. 1C), Badea Pancreas dataset (Fig. 1D) and Pei Pancreas dataset (Fig. 1E) showed the similar results. Besides, protein expression analysis by immunohistochemistry from The Human Protein Atlas (THPA) showed FEZ2 was highly expressed in PDAC tumor cells (strong intensity) compared with pancreas cells (moderate intensity) (Figs. 1F and 1G). Taken together, these data demonstrated the upregulation of FEZ2 in pancreatic cancer.

To understand the mechanism of FEZ2 upregulation in PDAC, we analyzed the correlation between FEZ2 mRNA expression with its copy number and DNA methylation. The results showed that FEZ2 expression was positive correlated with its copy number (Fig. 1H), while the methylation level on FEZ2 promoter region was negative correlated with its expression (Fig. 1I). These indicated that increased copy number and reduced methylation level might be the reason of FEZ2 upregulation in PDAC.

### FEZ2 methylation and prognosis of PDAC patients

**Figure 2:**
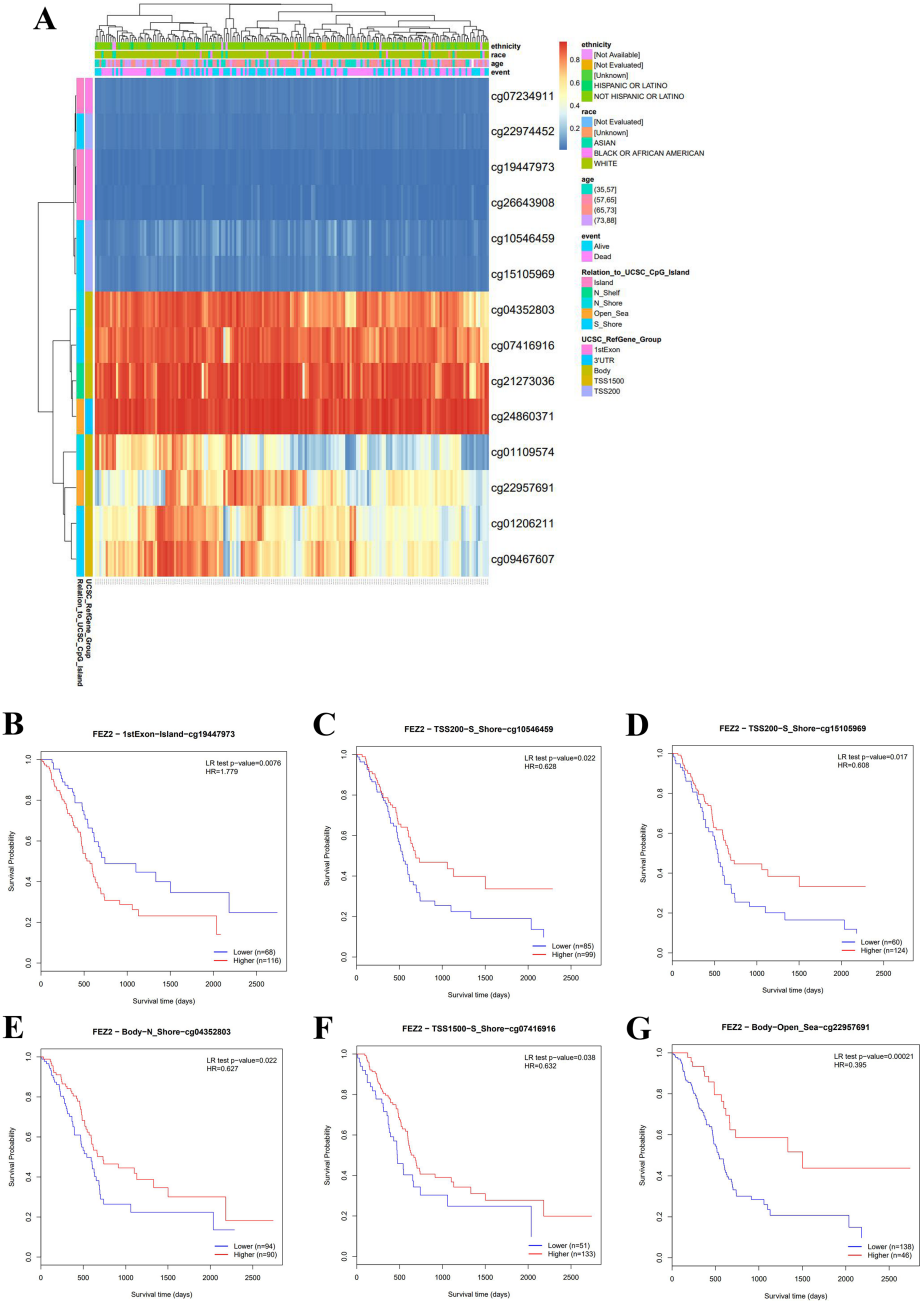
Methylation pattern of FEZ2 and survival analysis of its methylation sites. (A) Heatmap showed different methylation sites of FEZ2. (B–G) Survival analysis of different FEZ2 methylation sites.

To clarify the clinical value of FEZ2 methylation, we investigated the methylation pattern of different CpG sites on FEZ2 promoter region with clinical parameters including ethnicity, race, age and so on, the results were summarized as heatmap (Fig. 2A). Besides, survival analysis of different methylation sites showed that six independent methylation sites were significantly correlated with PDAC patients’ survival (Figs. 2B–2G). Among them, only one site showed that higher methylation predicted shorter survival time, other five sites suggested lower methylation predicted shorter survival time. Therefore, these methylation sites of FEZ2 might have clinical value for patient’s prognosis prediction. Since FEZ2 methylation was negatively correlated with its expression, High FEZ2 mRNA expression might be a risk factor for PDAC progression.

### FEZ2 was a risk factor for disease-free interval of PDAC patients and associated with 5-FU resistance

**Figure 3:**
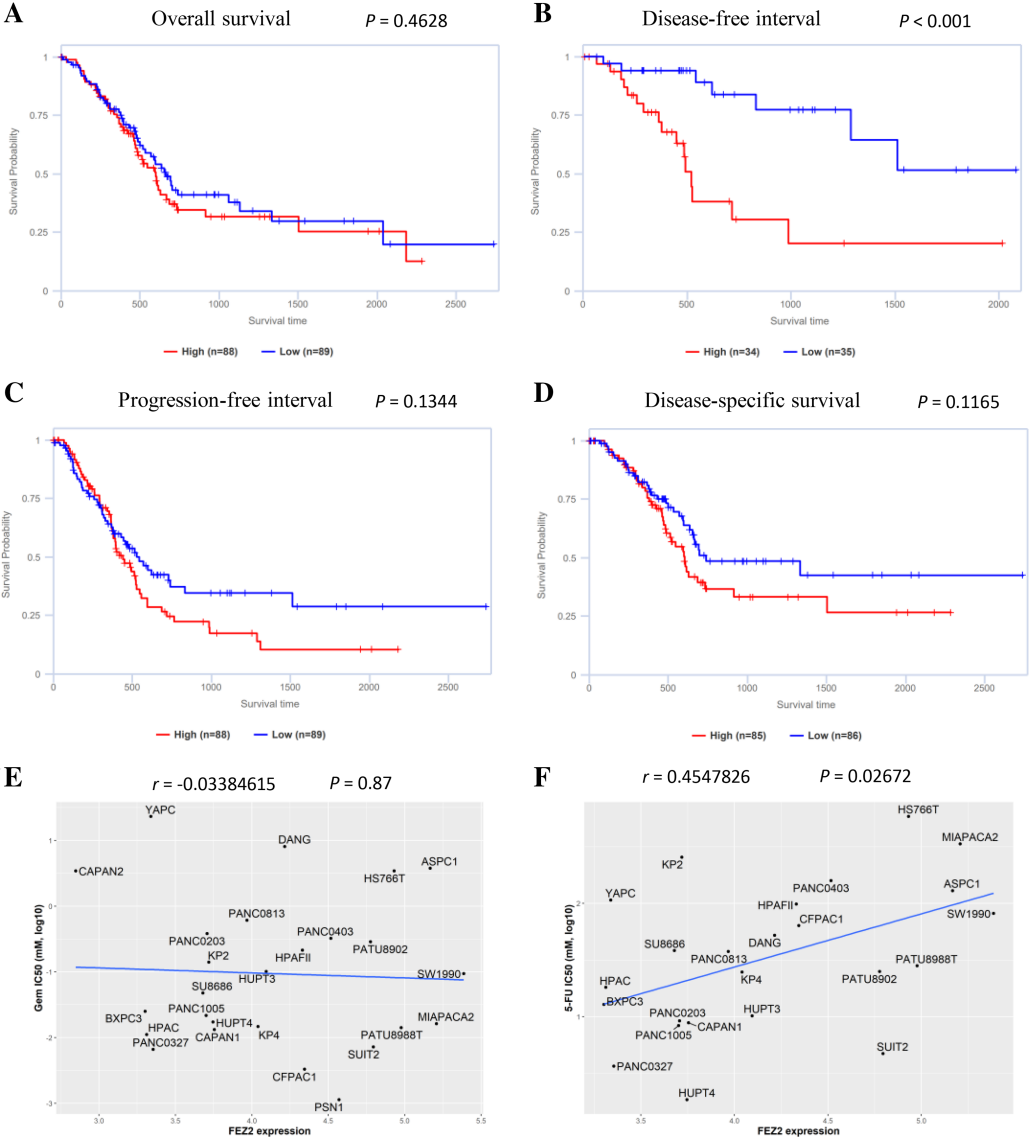
Association of FEZ2 expression with PDAC patient’s prognosis and chemotherapy drugs sensitivity. Kaplan-Meier plots showed the correlation between FEZ2 expression and overall survival (A), disease-free interval (B), progression-free interval (C) and disease-specific survival (D). Scatter plots performed the correlation between FEZ2 expression and half maximal inhibitory concentration (IC50) of Gemcitabine (E) and 5-FU (F).

To understand the role of FEZ2 on PDAC patient’s prognosis, we performed survival analysis based on TCGA database. As the results showed (Fig. 3B), the higher expression of FEZ2 predicted a significant shorter Disease-free interval. Other prognosis indicators including Overall survival (Fig. 3A), Progression-free interval (Fig. 3C) and Disease-specific interval (Fig. 3D) showed no significant correlation with FEZ2 expression. As disease-free interval can be usually used as an indicator to evaluate the effect of chemotherapy. This meant that high FEZ2 expression might promote tumor progression and chemotherapy resistance after surgery. To further determine the potential functions of FEZ2 on chemotherapy resistance, we explored the correlation between FEZ2 expression and IC50 of Gemcitabine and 5-FU in PDAC cell lines (Figs. 3E, 3F). As the results showed, IC50 of 5-FU was positively correlated with FEZ2 expression, but the IC50 of Gemcitabine showed no significant correlation with FEZ2. Collectively, these results indicated FEZ2 was a risk factor for disease-free interval of PDAC patients and might enhanced 5-FU resistance of PDAC cells.

### Association between FEZ2 and immune signatures

**Figure 4:**
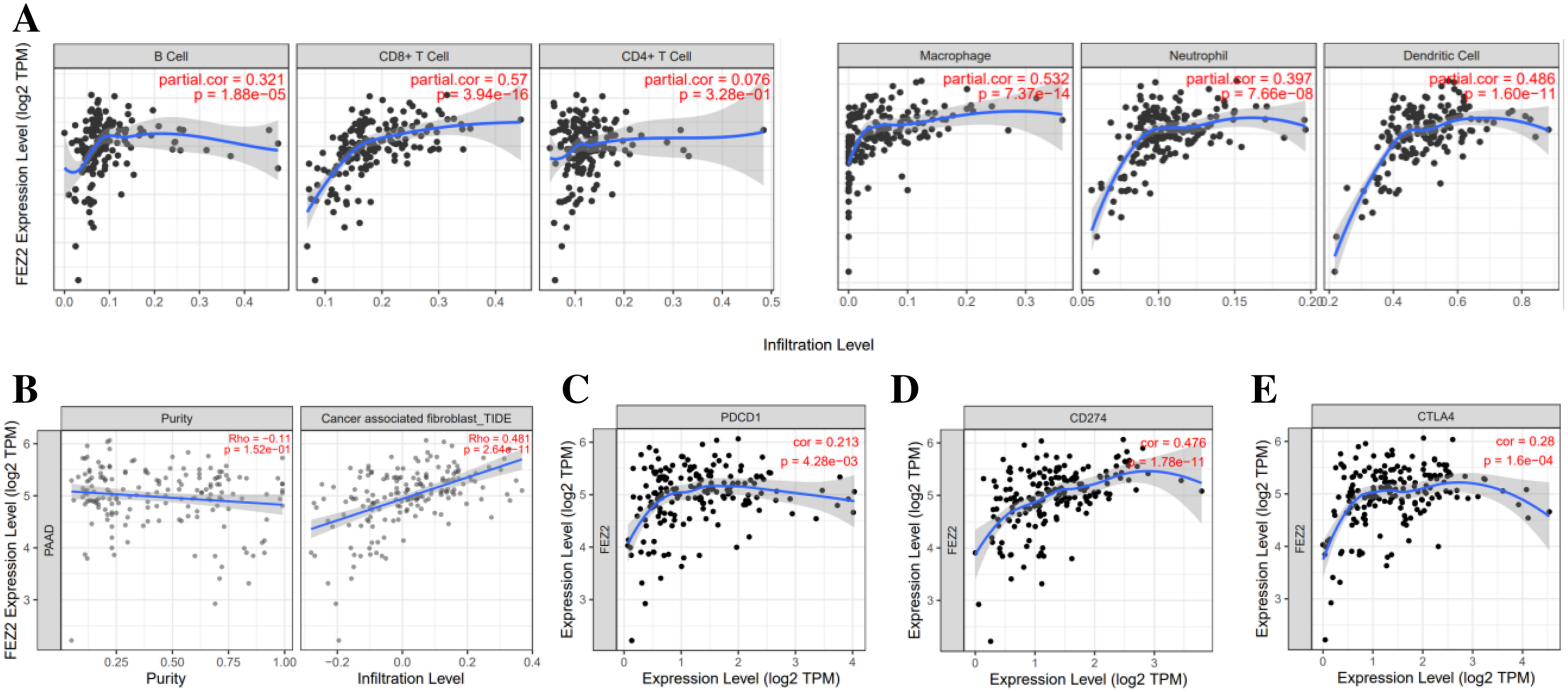
Relationship between FEZ2 expression and immune signatures. (A) Scatter plots showed the correlation between FEZ2 expression and different immune cell infiltration. (B) The association between FEZ2 expression and tumor purity and cancer associated fibroblast infiltration. The correlation between FEZ2 expression and immunotherapy markers including PDCD1 (C), CD274 (D) and CTLA4 (E).

To investigate the correlation between FEZ2 expression and immune cell infiltration, we used TIMER database to perform this analysis. As the results showed (Fig. 4A), FEZ2 expression was positive corelated with the infiltration of many immune cells including B cell (*r* = 0.321), CD8+ T cell (*r* = 0.57), macrophage (*r* = 0.532), neutrophil (*r* = 0.397) and dendritic cell (*r* = 0.486). In addition, we also focused on the correlation of FEZ2 with tumor purity and cancer associated fibroblast infiltration. As the results showed in Fig. 4B, FEZ2 was negatively correlated with tumor purity and positively correlated with cancer associated fibroblast infiltration. Moreover, because of the increasing expectation of immunotherapy, we further examined the correlation between FEZ2 expression and immunotherapy targets including PD-1 (PDCD1) (Fig. 4C), PD-L1 (CD274) (Fig. 4D) and CLTA4 (Fig. 4E). We found that these immunotherapy targets were positively correlated with FEZ2. Taken together, FEZ2 expression was positively correlated with immune cell infiltration, and increasing of cancer associated fibroblast infiltration might be a reason for chemotherapy resistance. Moreover, since FEZ2 was positively related to immunotherapy targets, immunotherapy might be a good choice for the patients with high FEZ2 expression.

### Association between FEZ2 expression and miRNA regulation

**Figure 5:**
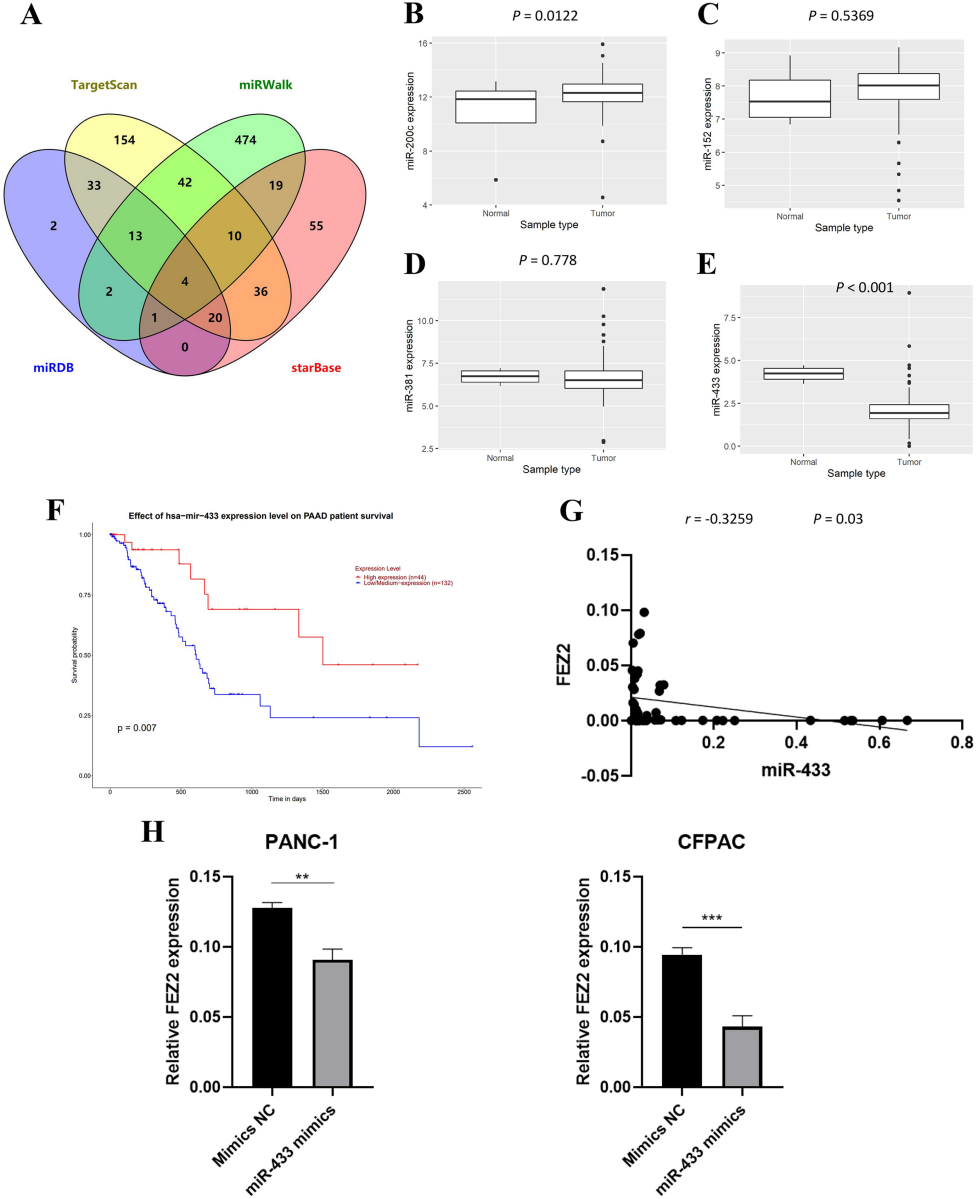
miRNA regulation of FEZ2 and its clinical value. (A) Venn plot showed the overlapping of FEZ2-targeted miRNAs. The expression of miRNAs based on TCGA database including miR-200c (B), miR-152 (C), miR-381 (D) and miR-433 (E). (F) Correlation of miR-433 expression with PDAC patients’ survival. (G) Scatter plot showed FEZ2 and miR-433 was negatively correlated in tumor tissues (*n* = 45, *r* = −0.3259, *P* = 0.03). (H) FEZ2 expression was significantly inhibited by miR-433-3p mimics compared with the mimic NC in PANC-1 and CFPAC cell lines. **P < 0.01, ***P < 0.001.

**Figure 6:**
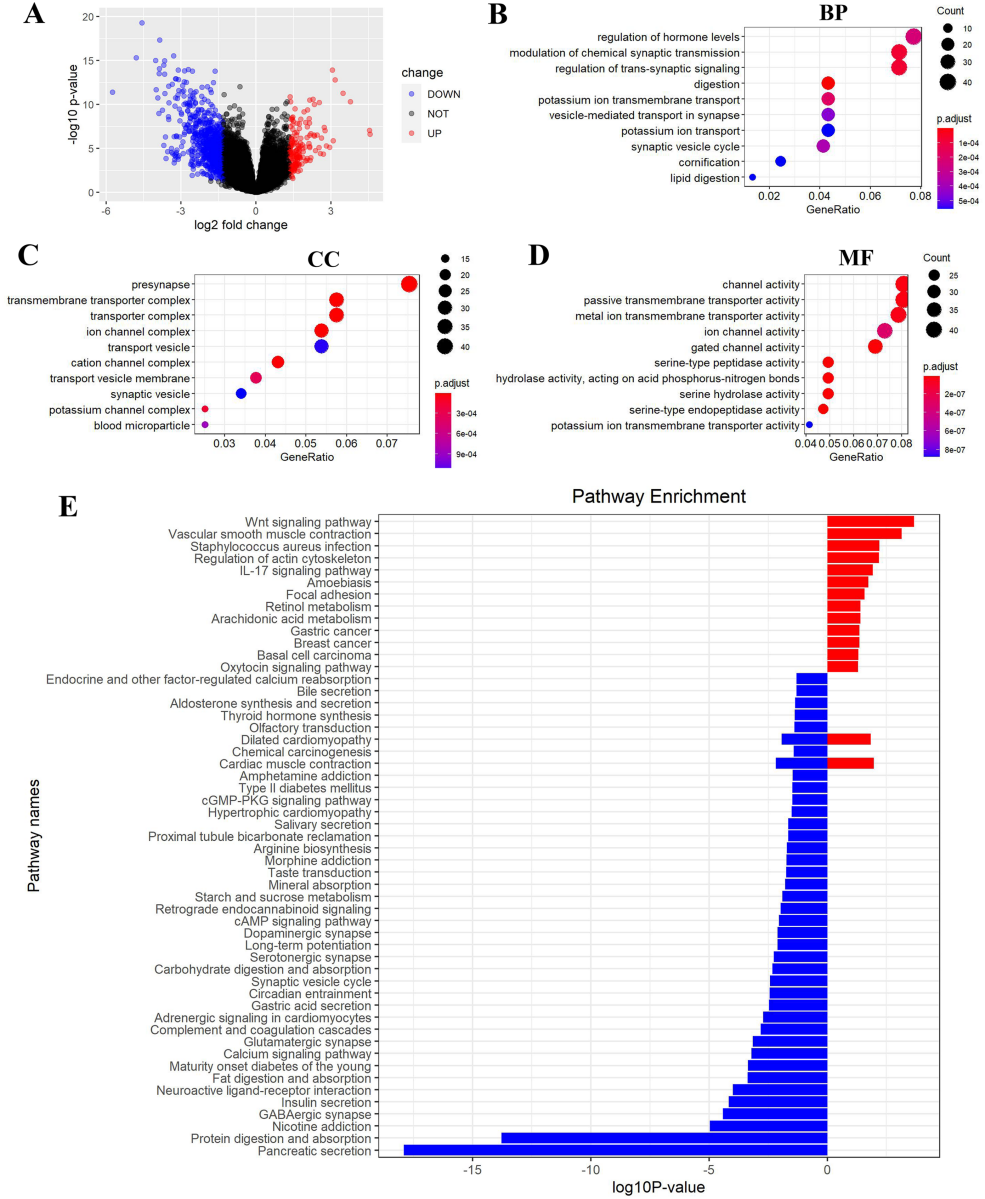
FEZ2-related pathways in PDAC. (A) Volcano plot showed DEGs (differently expressed genes) between FEZ2 high expression group and low expression group. Genes with |logFC| > 1.326 combined with *P* < 0.05 were identified as significant. We found 162 upregulated genes and 748 downregulated genes. GO (gene ontology) showed these DEGs were enriched in different biological process (B), cellular component (C) and molecular function (D). (E) GSEA (Gene set enrichment analysis) revealed the pathway enrichment of these DEGs.

To further investigate the molecular mechanism of FEZ2 upregulation in PDAC, we identified FEZ2-targeted miRNAs through multiple tools prediction including TargetScan, miRWalk, miRDB and starBase (Fig. 5A). Four miRNAs were identified by overlapping prediction results from different tools, including miR-200c, miR-152, miR-381 and miR-433. Boxplots revealed the expression of miRNA based on TCGA database (Figs. 5B–5E). Among them, only miR-433 was significantly low expressed in PDAC tissues. In addition, the patients with low miR-433 expression predicted a poor prognosis (Fig. 6F). The results revealed that FEZ2 and miR-433 was negatively correlated in tumor tissues (*n* = 45, *r* = −0.3259, *P* = 0.03) (Fig. 5G). As expected, the FEZ2 expression was significantly inhibited by miR-433-3p mimics compared with the mimic NC (Fig. 5H). This suggested that miR-433 downregulation might upregulated FEZ2 in PDAC *via* ceRNA mechanism.

### FEZ2 regulated signaling pathways in PDAC

To gain insight into the function of FEZ2 in PDAC at the transcriptome level, we divided the PDAC patients into FEZ2 higher expression group and FEZ2 lower expression group from TCGA database. DEGs were selected by |logFC| > 1.326 combined with *P* < 0.05. As the volcano plot showed (Fig. 6A), finally we got 162 upregulated genes and 748 downregulated genes. Gene Ontology showed these DEGs were enriched in terms of Biological Process (Fig. 6B), Cellular Component (Fig. 6C) and Molecular Function (Fig. 6D). Specially, as the results showed in Fig. 6B, FEZ2 was involved in biological process including regulation of hormone levels, modulation of chemical synaptic transmission and regulation of trans-synaptic signaling. Further gene pathway enrichment analysis revealed that over-representative suppressed pathways included pancreatic secretion, protein digestion and absorption, indicating that the mis-regulation of FEZ2 may cause losing normal digestive functions of the pancreas cells. In contrast, the most representative active pathway was Wnt signaling pathway (Fig. 6E), which was proved to promote the progression of many cancers. These results suggested that FEZ2 might promote PDAC progression through Wnt signaling.

### FEZ2 promoted proliferation and migration in PDAC cell lines

**Figure 7:**
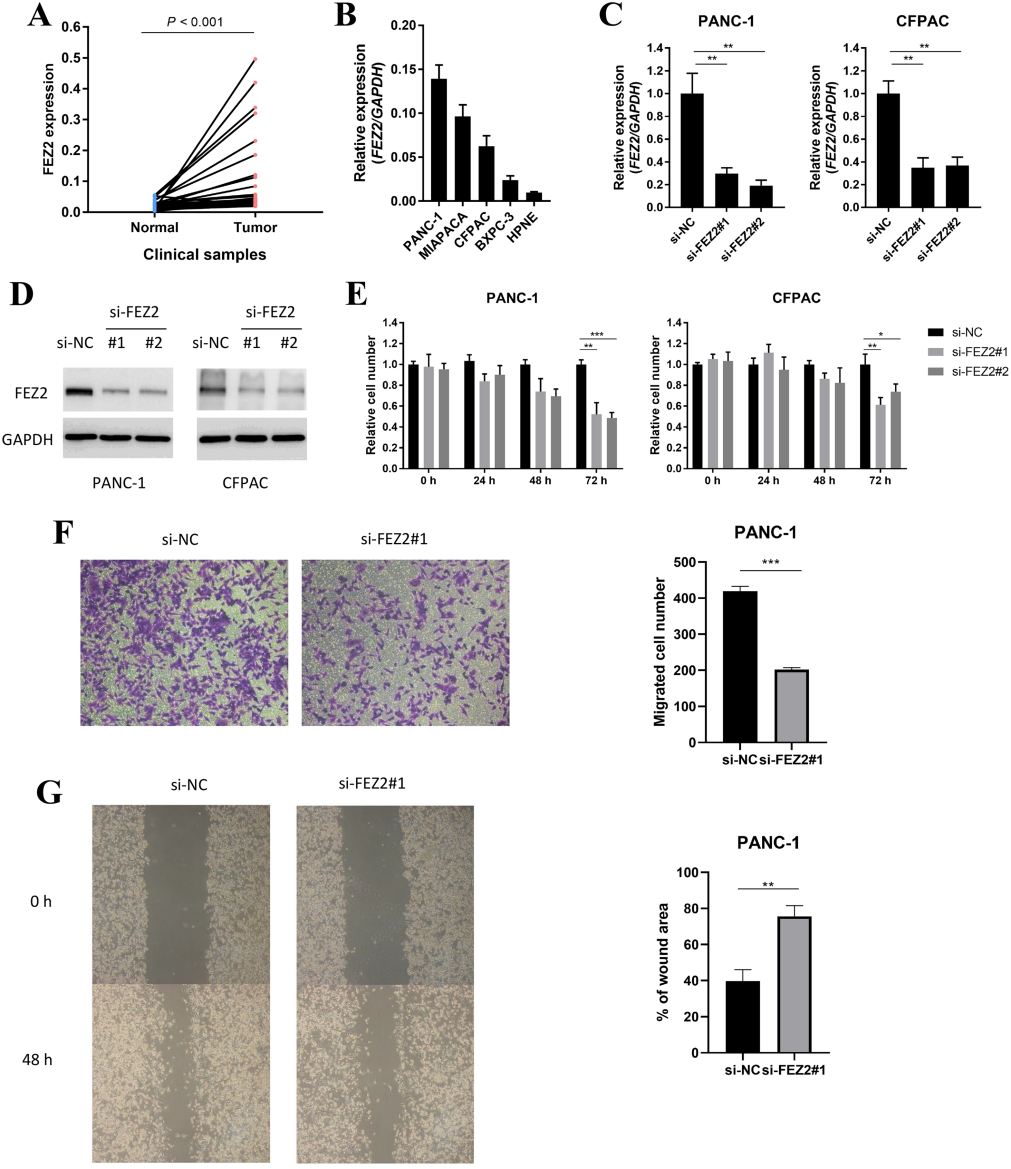
FEZ2 promoted proliferation and migration in PDAC cell lines. (A) FEZ2 expression in clinical samples. (B) qPCR showed the expression of FEZ2 in PDAC cell lines. (C) FEZ2 mRNA expression in PANC-1 (left) and CFPAC (right) cells after knockdown by two different siRNA. (D) Western blot showed FEZ2 protein expression in PANC-1 (left) and CFPAC (right) cells after siRNA knockdown. (E) Effects of si-FEZ2 #1 and #2 on proliferation of PANC-1 (left) and CFPAC (right) cells. The x axis indicates hours after siRNA treatment, the y axis indicates cell number relative to si-NC group. Transwell assay (F) and wound-healing assay (G) showed the migration of PANC-1 cells were inhibited by si-FEZ2#1, the data was quantified by ImageJ and showed significant difference. *P < 0.05, **P < 0.01, ***P < 0.001.

To validated the potential oncogenic functions of FEZ2, we validated the expression and functions of FEZ2 in PDAC cell lines and our clinical samples. First, we examined the expression of FEZ2 mRNA in PDAC tissues (*n* = 40) and adjacent normal tissues (*n* = 40), and found FEZ2 was significantly upregulated in our PDAC tissues (Fig. 7A). Then, we compared FEZ2 expression in different PDAC cell lines (Fig. 7B). PANC-1 and CFPAC were chosen for the later experiments. FEZ2 was silenced in PANC-1 and CFPAC cell lines by using two different siRNAs (siFEZ2#1 and siFEZ2#2). Both siRNA #1 and #2 showed > 50% depletion of FEZ2 expression in these two cell lines (Fig. 7C), and the corresponding FEZ2 protein levels examined by Western blot were consistent with the mRNA levels (Fig. 7D). As shown in Fig. 7E, although there was no much differences in 24 h, the depletion of FEZ2 in siRNA#2 and #3 significantly suppressed the proliferation of PANC-1 and CFPAC cell lines after 72 h. Transwell assay (Fig. 7F) and wound-healing assay (Fig. 7G) showed the migration of PANC-1 cells were inhibited by si-FEZ2#1.

### FEZ2 promoted 5-FU resistance and Wnt signaling activation in PDAC cell lines

**Figure 8:**
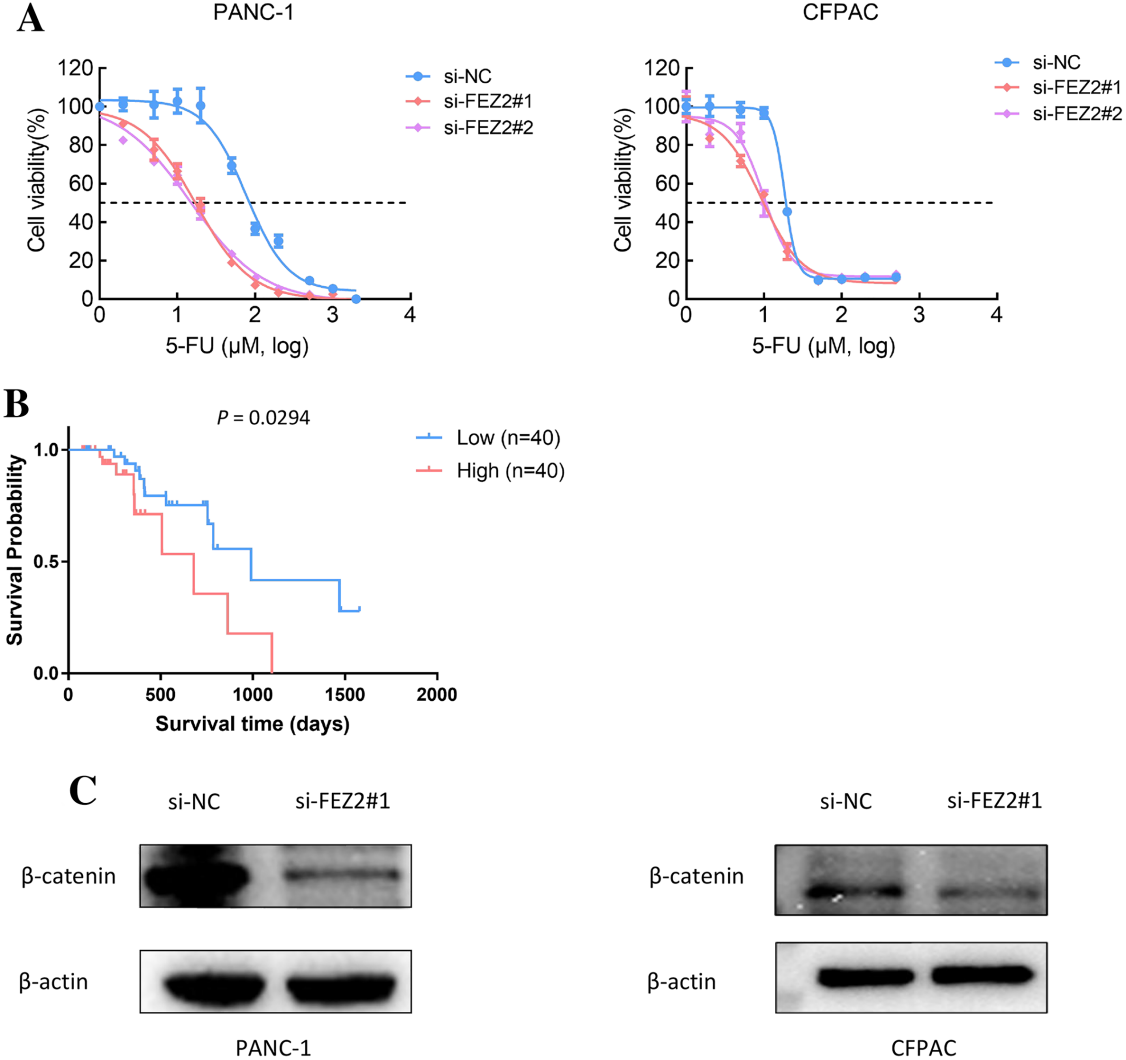
FEZ2 promoted 5-FU resistance and Wnt signaling activation in PDAC cell lines. (A) Dose response curve of 5-FU in PANC-1 (left) and CFPAC (right) cells after transfected with si-NC or si-FEZ2. (B) Kaplan-Meier plot showed the effect of FEZ2 expression on the overall survival rate of patients who received 5-FU chemotherapy (High group, *n* = 40; Low group, *n* = 40). (C) Western blotting showed key protein expression of Wnt/β-catenin was inhibited by FEZ2 siRNA in PANC-1 and CFPAC cell lines.

As 5-FU is one of the first-line chemotherapeutic agents [4], to further validate the potential functions of FEZ2 on 5-FU resistance, we performed the drug sensitivity assay by using PDAC cell lines. As the results showed, depletion of FEZ2 sensitized PANC-1 and CFPAC cell lines to 5-FU treatment displaying by decreased cell viabilities (Fig. 8A). In addition, by comparing FEZ2 expression and overall survival rate of PDAC patients who received 5-FU chemotherapy (*n* = 80), we found that higher FEZ2 expression was correlated with shorter overall survival time (Fig. 8B). To examine the intracellular signaling mechanism between FEZ2 and Wnt/β-catenin signaling pathway followed by western blotting. As shown in (Fig. 8C), the expression of cytoplasmic β-catenin was significantly inhibited by si-FEZ2. Collectively, these results indicated FEZ2 enhanced 5-FU resistance and Wnt signaling activation in PDAC cell lines.

## Discussion

Although FEZ2 is dysregulated in some types of cancers, there is no research mentioned that FEZ2 functions as an oncogene or tumor suppressor gene in PDAC. As the member of FEZ family, FEZ1 has been more studied compared with FEZ2. FEZ1 could regulate axons formation during development, but the function of FEZ2 is unclear yet (Fujita et al., 2004; Gindhart et al., 2003). Recent study showed that although FEZ2 shared conserved region with FEZ1 at their C-terminal (Bloom & Horvitz, 1997; Fujita et al., 2004; Kuroda et al., 1999), FEZ2 acquired additional protein interaction patterns during evolution (Alborghetti, Furlan & Kobarg, 2011). FEZ2 interacted proteins existed both in nucleus and cytoplasm, indicating FEZ2 could be involved in cell function regulation in multiple ways. In the current study, we uncovered the potential oncogenic effects of FEZ2 on PDAC. FEZ2 depletion by siRNA resulted in inhibition of proliferation, migration and 5-FU resistance of PDAC cells. Wnt signaling activation was significantly correlated with FEZ2 expression levels in PDAC, FEZ2 depletion results in suppression of β-catenin expression.

By mining two large-scale database, TCGA and GTEx, we revealed that FEZ2 was highly expressed in PDAC tissues, suggested the function of FEZ2 was required in PDAC occurrence and development. Immunohistochemistry from THPA database showed that FEZ2 staining had a moderate intensity in normal pancreatic cells in contrast to a strong intensity in PDAC cells. Our clinical specimens also validated the upregulation of FEZ2 on PDAC. Besides, FEZ2 expression was negatively correlated with its methylation, and both low expression and high methylation of FEZ2 predicted a favorable prognosis. Further silencing of FEZ2 significantly decreased PDAC cell proliferation. These results collectively suggested the function of FEZ2 was required in PDAC occurrence and development.

Recently, a lot of studies pointed that aberrant DNA methylation was one of the important biomarkers for PDAC diagnosis and prognosis (Henriksen et al., 2015; Natale et al., 2019; Warton & Samimi, 2015). In this study, we identified the negative correlation between FEZ2 methylation and its expression. This negative correlation suggested the mechanism of high FEZ2 expression in PDAC. To investigate the prognostic value of FEZ methylation, we further analyzed different CpG sites on FEZ2 promoter region. Six CpG sites were identified to be significantly correlated with the overall survival rates of PDAC patients. Among them, five CpG sites showed that high methylation associated with favorable survival rates. Collectively, FEZ2 expression was negatively regulated by its methylation, and FEZ2 methylation might be a potential marker for PDAC patients’ survival.

Also, downregulation of miR-433 might be a reason for upregulation of FEZ2 in PDAC. Database prediction showed that FEZ2 might be regulated by miR-433 by a competing endogenous RNA manner. In the 3′UTR of FEZ2 mRNA, miR-433 seed sequence was identified, and miR-433 could inhibit FEZ2 mRNA expression by recruiting RNA-induced silencing complex (RISC). To validate this regulation mechanism, we introduced miR-433 mimics in PDAC cell lines, and the mimics successfully suppressed FEZ2 mRNA expression. Clinical samples also suggested the negative correlation between FEZ2 and miR-433.

Besides, FEZ2 expression was also correlated with PDAC patient’s prognosis. Clinical information of the patients from TCGA database showed that higher FEZ2 expression was associated with shorter disease-free interval. Since disease-free interval was usually used as an indicator to evaluate the treatment outcome of chemotherapy, so patients with high FEZ2 expression were considered to have more recurrence and bad prognosis. While chemotherapy resistance was one of the main reasons to cause relapse, we were altered to investigate the possible effects of FEZ2 on the chemosensitivity. The result clearly showed the depletion of FEZ2 sensitized 5-FU treatment of PDAC cells *in vitro*, and high FEZ2 expression of PDAC patients who received 5-FU chemotherapy had a poor prognosis. Therefore, FEZ2 could be used as a marker to guide the chemotherapy drug choice for the PDAC patients.

Immunotherapy has attracted a lot of researchers’ attentions in recent years, and it has become one of the promising therapies for PDAC (Aroldi & Zaniboni, 2017; Balachandran, Beatty & Dougan, 2019; Feng et al., 2017; Morrison, Byrne & Vonderheide, 2018). In the present study, we observed that FEZ2 expression was positively correlated with many immune cells including macrophage, neutrophil, dendritic cell, B cell and CD8+ T cell. Then, we analyzed the association between FEZ2 and immunotherapy targets including PD-1, PD-L1 and CLTA4, results showed that these targets were positively correlated with FEZ2. This indicated that patients with high FEZ2 expression were sensitive to the immunotherapy. Also, we found that FEZ2 was positively related to cancer associated fibroblast. Recent studies showed that cancer associated fibroblast was one of the reasons for chemotherapy resistance (Han et al., 2020; Pan et al., 2015; von Ahrens et al., 2017). This explained the potential mechanism for FEZ2 on 5-FU resistance. Taken together, FEZ2 was positively related to immune cell infiltration in PDAC, and patients with high FEZ2 expression were predicted to be sensitive to immunotherapy targeting PD-1, PD-L1 and CLTA4.

After revealing the oncogenic effects and prognostic value of FEZ2, we tried to understand the functional mechanism and regulation network of FEZ2. Subsequent gene pathway enrichment analysis of the co-expressed genes in FEZ2 high expression patients showed the most significant activated pathway was Wnt signaling, suggesting that FEZ2 might promote PDAC cell proliferation and 5-FU resistance through activation of Wnt signaling. Western blot showed the key protein expression of Wnt/β-catenin was inhibited by FEZ2 siRNA in PANC-1 and CFPAC cell lines. Although the Wnt signaling pathway was involved in many cancer progressions, its function in PDAC and the role of FEZ2 within this pathway need to be studied further.

## Conclusions

In conclusion, our data provided the evidence that FEZ2 functioned as an oncogenic gene in PDAC by migration, promoting cell proliferation and chemotherapy resistance, the expression and methylation level of FEZ2 had prognostic value for PDAC patients. Although further study is required to illustrate the detailed mechanism of FEZ2 on PDAC progression, our data provide a new target for both chemotherapy program guiding and therapeutic treatment targeting.

## Supplemental Information

10.7717/peerj.12736/supp-1 Supplemental Information 1 Different expression gene.

10.7717/peerj.12736/supp-2 Supplemental Information 2 Kyoto Encyclopedia of genes and genomes.

10.7717/peerj.12736/supp-3 Supplemental Information 3 Identification of FEZ2 as a novel oncogene in pancreatic ductal adenocarcinoma.

10.7717/peerj.12736/supp-4 Supplemental Information 4 Western blot.

10.7717/peerj.12736/supp-5 Supplemental Information 5 TCGA pancreatic cancer data.

10.7717/peerj.12736/supp-6 Supplemental Information 6 Cell line experiment.

10.7717/peerj.12736/supp-7 Supplemental Information 7 FEZ2 expression in tissues.

10.7717/peerj.12736/supp-8 Supplemental Information 8 Clinical tissue samples. 50 pairs tissues

10.7717/peerj.12736/supp-9 Supplemental Information 9 Cell line expression.

10.7717/peerj.12736/supp-10 Supplemental Information 10 Logsdon panc.

10.7717/peerj.12736/supp-11 Supplemental Information 11 Pei pancreas.

10.7717/peerj.12736/supp-12 Supplemental Information 12 Badea Pancreas.

10.7717/peerj.12736/supp-13 Supplemental Information 13 Survival times FEZ2.

10.7717/peerj.12736/supp-14 Supplemental Information 14 CFPAC mimics.

10.7717/peerj.12736/supp-15 Supplemental Information 15 Western blot of β-catenin.

10.7717/peerj.12736/supp-16 Supplemental Information 16 TCGA GTEx FEZ2 expression.

10.7717/peerj.12736/supp-17 Supplemental Information 17 Tissue correlation.

10.7717/peerj.12736/supp-18 Supplemental Information 18 CFPAC cell proliferation. NC
siFEZ2#1
siFEZ2#2

10.7717/peerj.12736/supp-19 Supplemental Information 19 PANC-1 mimics.

10.7717/peerj.12736/supp-20 Supplemental Information 20 CFPAC ic50. NC
siFEZ2#1
siFEZ2#2

10.7717/peerj.12736/supp-21 Supplemental Information 21 CFPAC-si. Cfpac-fez2 knock down

10.7717/peerj.12736/supp-22 Supplemental Information 22 PANC-1 ic50.

10.7717/peerj.12736/supp-23 Supplemental Information 23 PANC-1 cell expression in three groups. NC
si-FEZ2#1
si-FEZ2#2

10.7717/peerj.12736/supp-24 Supplemental Information 24 PANC-1 FEZ2 knockdown. NC si-FEZ2#1 si-FEZ2#2

## Abbreviations

PDAC: pancreatic ductal adenocarcinoma
FEZ2: Fasciculation and Elongation protein Zeta 2
TCGA: The Cancer Genome Atlas
5-FU: 5-Fluorouracil
PPI: protein-protein interactions
GSEA: Gene set enrichment analysis
CCLE: Cancer Cell Line Encyclopedia
GDSC: Genomics of Drug Sensitivity in Cancer
DEGs: differently expressed genes
GO: Gene Ontology
KEGG: Kyoto Encyclopedia of Genes and Genomes
THPA: The Human Protein Atlas

## Additional Information and Declarations

### Data Availability

The following information was supplied regarding data availability: The raw measurements are available in the Supplemental Files.
