# Supplementary material for: Identification of FEZ2 as a potential oncogene in pancreatic ductal adenocarcinoma
Source: PeerJ. 2022 Jan 5;10:e12736. doi: 10.7717/peerj.12736 (PMC8742541; doi:10.7717/peerj.12736)
Supplement: Supplemental Information 3 [file peerj-10-12736-s003.pdf]

Figure 1

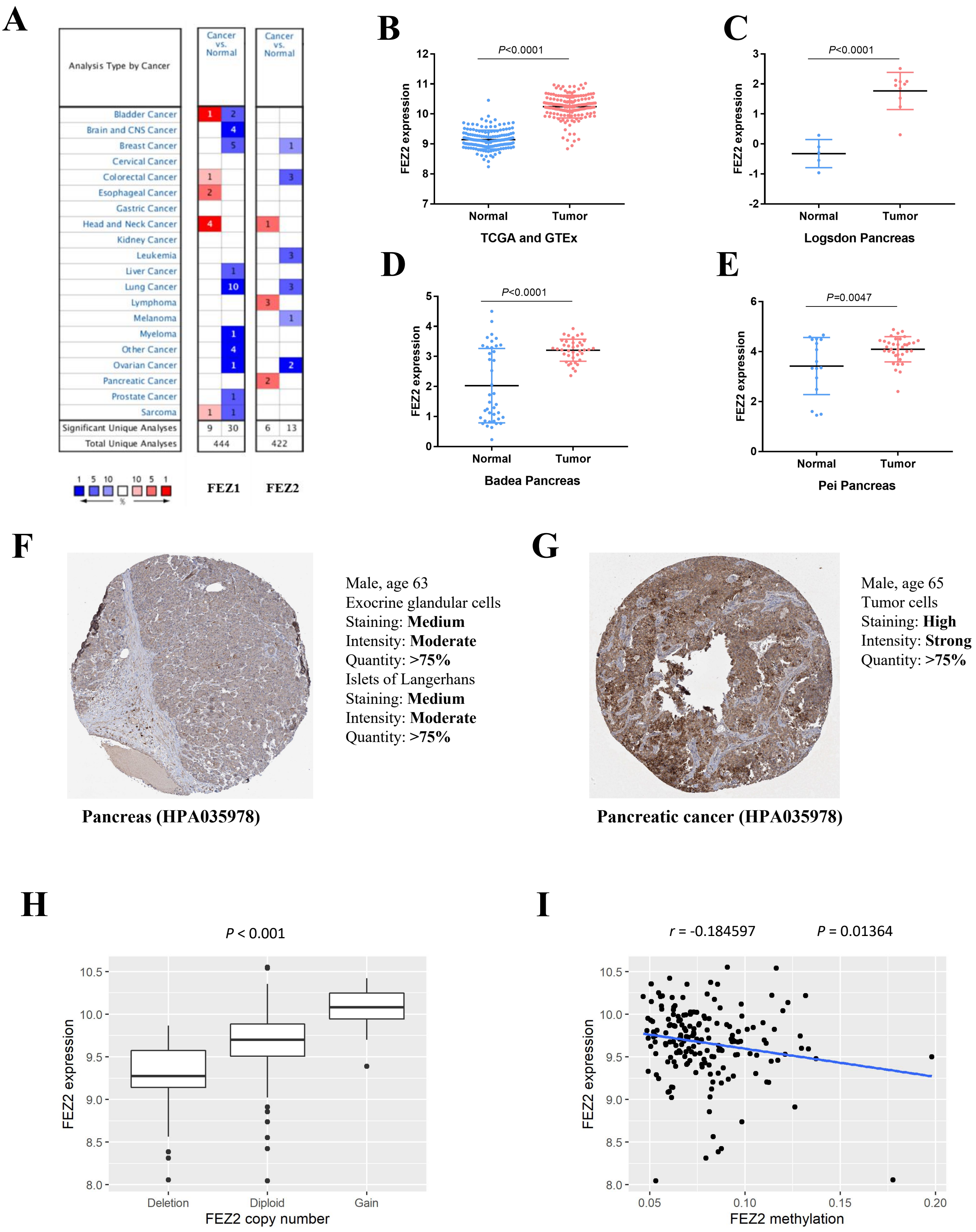
A
Analysis Type by Cancer
Cancer vs. Normal
Bladder Cancer
Brain and CNS Cancer
Breast Cancer
Cervical Cancer
Colorectal Cancer
Esophageal Cancer
Gastric Cancer
Head and Neck Cancer
Kidney Cancer
Leukemia
Liver Cancer
Lung Cancer
Lymphoma
Melanoma
Myeloma
Other Cancer
Ovarian Cancer
Pancreatic Cancer
Prostate Cancer
Sarcoma
Significant Unique Analyses
Total Unique Analyses
FEZ1
FEZ2
B
P<0.0001
FEZ2 expression
Normal
Tumor
TCGA and GTEx
C
P<0.0001
Logsdon Pancreas
D
P<0.0001
Badea Pancreas
E
P=0.0047
Pei Pancreas
F
Male, age 63
Exocrine glandular cells
Staining: Medium
Intensity: Moderate
Quantity: >75%
Islets of Langerhans
Staining: Medium
Intensity: Moderate
Quantity: >75%
Pancreas (HPA035978)
G
Male, age 65
Tumor cells
Staining: High
Intensity: Strong
Quantity: >75%
Pancreatic cancer (HPA035978)
H
P < 0.001
FEZ2 expression
Deletion
Diploid
Gain
FEZ2 copy number
I
r = -0.184597
P = 0.01364
FEZ2 methylation

Figure 2

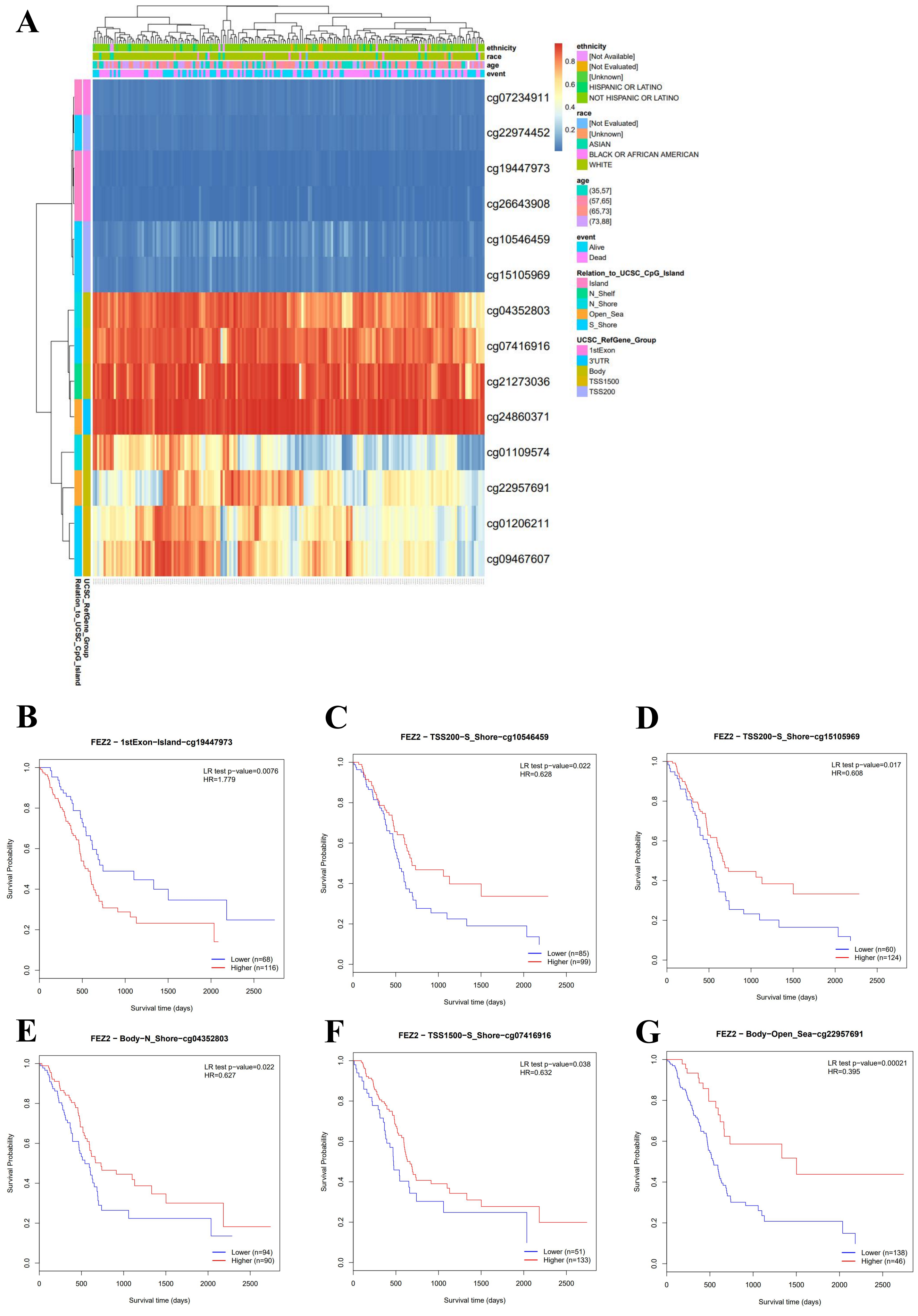

A
ethnicity
race
age
event
cg07234911
cg22974452
cg19447973
cg26643908
cg10546459
cg15105969
cg04352803
cg07416916
cg21273036
cg24860371
cg01109574
cg22957691
cg01206211
cg09467607
UCSC_RefGene_Group
Relation_to_UCSC_CpG_Island
0.8
0.6
0.4
0.2
ethnicity
[Not Available]
[Not Evaluated]
[Unknown]
HISPANIC OR LATINO
NOT HISPANIC OR LATINO
race
[Not Evaluated]
[Unknown]
ASIAN
BLACK OR AFRICAN AMERICAN
WHITE
age
(35,57]
(57,65]
(65,73]
(73,88]
event
Alive
Dead
Relation_to_UCSC_CpG_Island
Island
N_Shelf
N_Shore
Open_Sea
S_Shore
UCSC_RefGene_Group
1stExon
3'UTR
Body
TSS1500
TSS200
B
FEZ2 – 1stExon–Island–cg19447973
LR test p–value=0.0076
HR=1.779
Lower (n=68)
Higher (n=116)
Survival Probability
Survival time (days)
C
FEZ2 – TSS200–S_Shore–cg10546459
LR test p–value=0.022
HR=0.628
Lower (n=85)
Higher (n=99)
D
FEZ2 – TSS200–S_Shore–cg15105969
LR test p–value=0.017
HR=0.608
Lower (n=60)
Higher (n=124)
E
FEZ2 – Body–N_Shore–cg04352803
LR test p–value=0.022
HR=0.627
Lower (n=94)
Higher (n=90)
F
FEZ2 – TSS1500–S_Shore–cg07416916
LR test p–value=0.038
HR=0.632
Lower (n=51)
Higher (n=133)
G
FEZ2 – Body–Open_Sea–cg22957691
LR test p–value=0.00021
HR=0.395
Lower (n=138)
Higher (n=46)

Figure 3

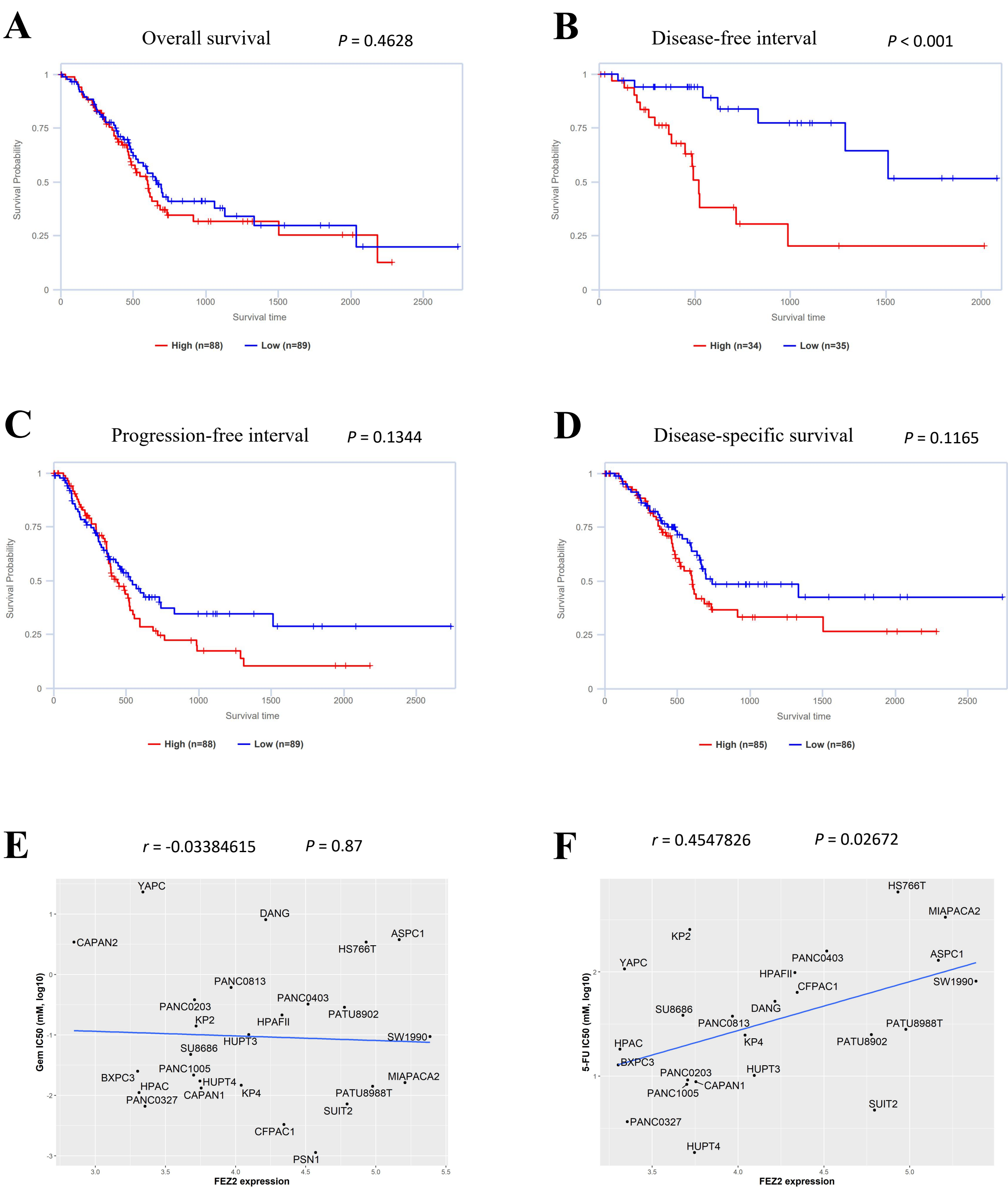

A
Overall survival
P = 0.4628
Survival Probability
Survival time
High (n=88)
Low (n=89)
B
Disease-free interval
P < 0.001
Survival Probability
Survival time
High (n=34)
Low (n=35)
C
Progression-free interval
P = 0.1344
Survival Probability
Survival time
High (n=88)
Low (n=89)
D
Disease-specific survival
P = 0.1165
Survival Probability
Survival time
High (n=85)
Low (n=86)
E
r = -0.03384615
P = 0.87
Gem IC50 (mM, log10)
FEZ2 expression
YAPC
DANG
CAPAN2
ASPC1
HS766T
PANC0813
PANC0403
PANC0203
PATU8902
KP2
HPAFII
SW1990
HUPT3
SU8686
PANC1005
BXPC3
HUPT4
MIAPACA2
HPAC
KP4
PATU8988T
PANC0327
CAPAN1
SUIT2
CFPAC1
PSN1
F
r = 0.4547826
P = 0.02672
5-FU IC50 (mM, log10)
FEZ2 expression
HS766T
MIAPACA2
KP2
PANC0403
ASPC1
YAPC
HPAFII
SW1990
CFPAC1
SU8686
DANG
PANC0813
PATU8988T
HPAC
KP4
PATU8902
BXPC3
PANC0203
HUPT3
CAPAN1
PANC1005
SUIT2
PANC0327
HUPT4

Figure 4

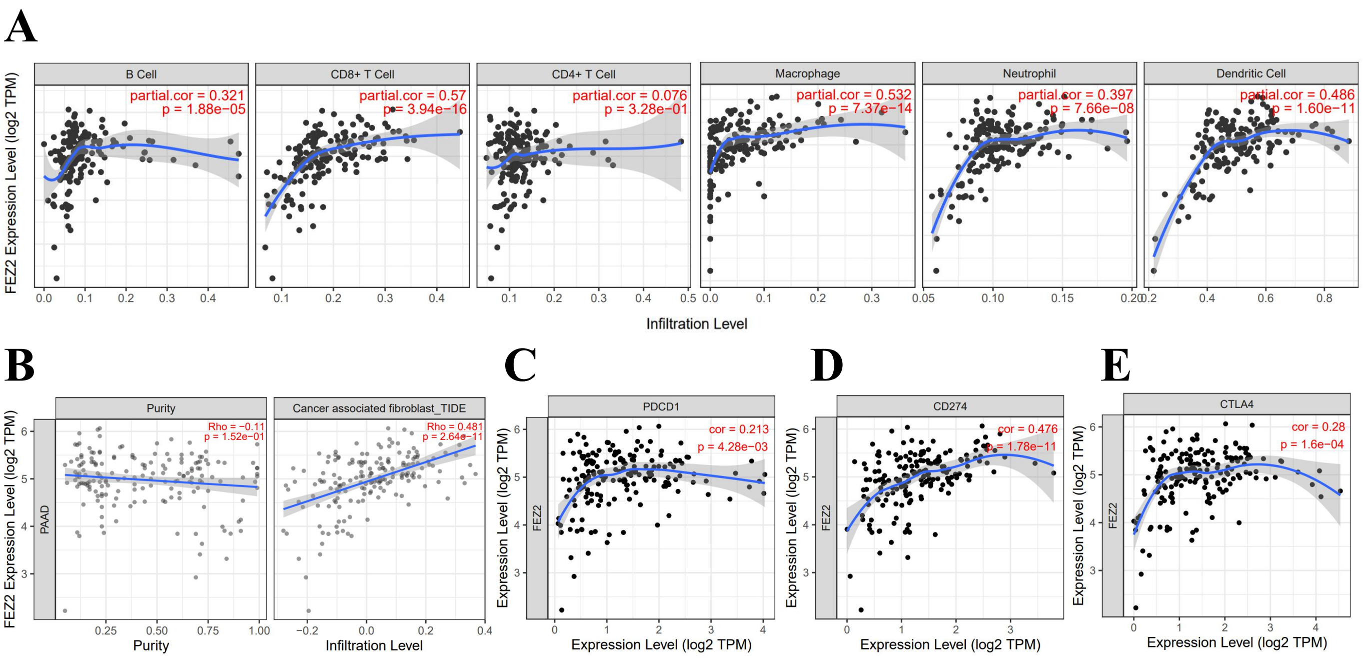
A
B Cell
partial.cor = 0.321
p = 1.88e−05
CD8+ T Cell
partial.cor = 0.57
p = 3.94e−16
CD4+ T Cell
partial.cor = 0.076
p = 3.28e−01
Macrophage
partial.cor = 0.532
p = 7.37e−14
Neutrophil
partial.cor = 0.397
p = 7.66e−08
Dendritic Cell
partial.cor = 0.486
p = 1.60e−11
FEZ2 Expression Level (log2 TPM)
Infiltration Level
B
Purity
Rho = −0.11
p = 1.52e−01
Cancer associated fibroblast_TIDE
Rho = 0.481
p = 2.64e−11
PAAD
FEZ2 Expression Level (log2 TPM)
Purity
Infiltration Level
C
PDCD1
cor = 0.213
p = 4.28e−03
D
CD274
cor = 0.476
p = 1.78e−11
E
CTLA4
cor = 0.28
p = 1.6e−04
FEZ2
Expression Level (log2 TPM)

Figure 5

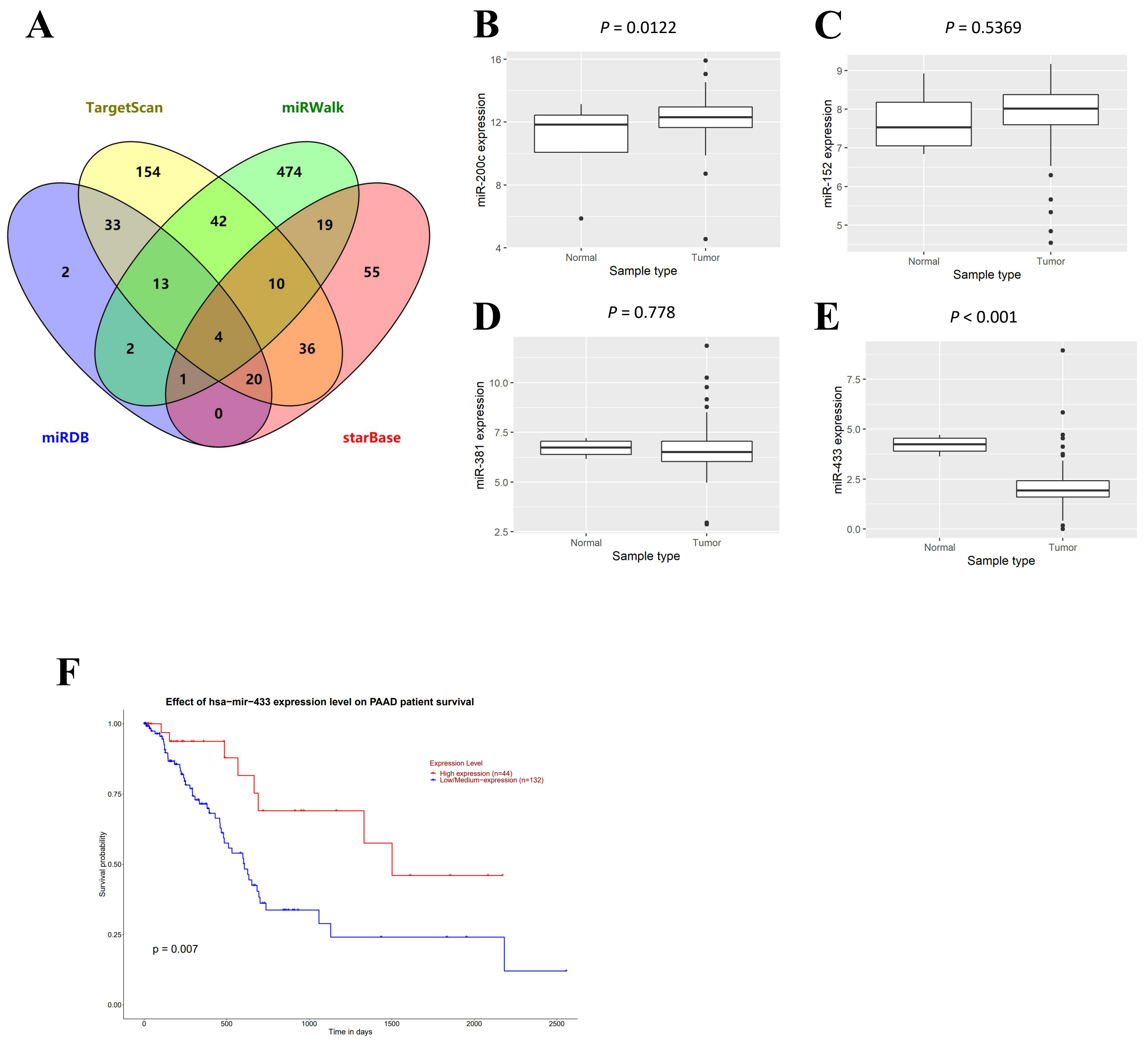
A
TargetScan
miRWalk
miRDB
starBase
154
474
33
42
19
2
13
10
55
4
2
36
1
20
0
B
P = 0.0122
miR-200c expression
Sample type
Normal
Tumor
C
P = 0.5369
miR-152 expression
Sample type
Normal
Tumor
D
P = 0.778
miR-381 expression
Sample type
Normal
Tumor
E
P < 0.001
miR-433 expression
Sample type
Normal
Tumor
F
Effect of hsa−mir−433 expression level on PAAD patient survival
Expression Level
High expression (n=44)
Low/Medium−expression (n=132)
Survival probability
Time in days
p = 0.007

Figure 6

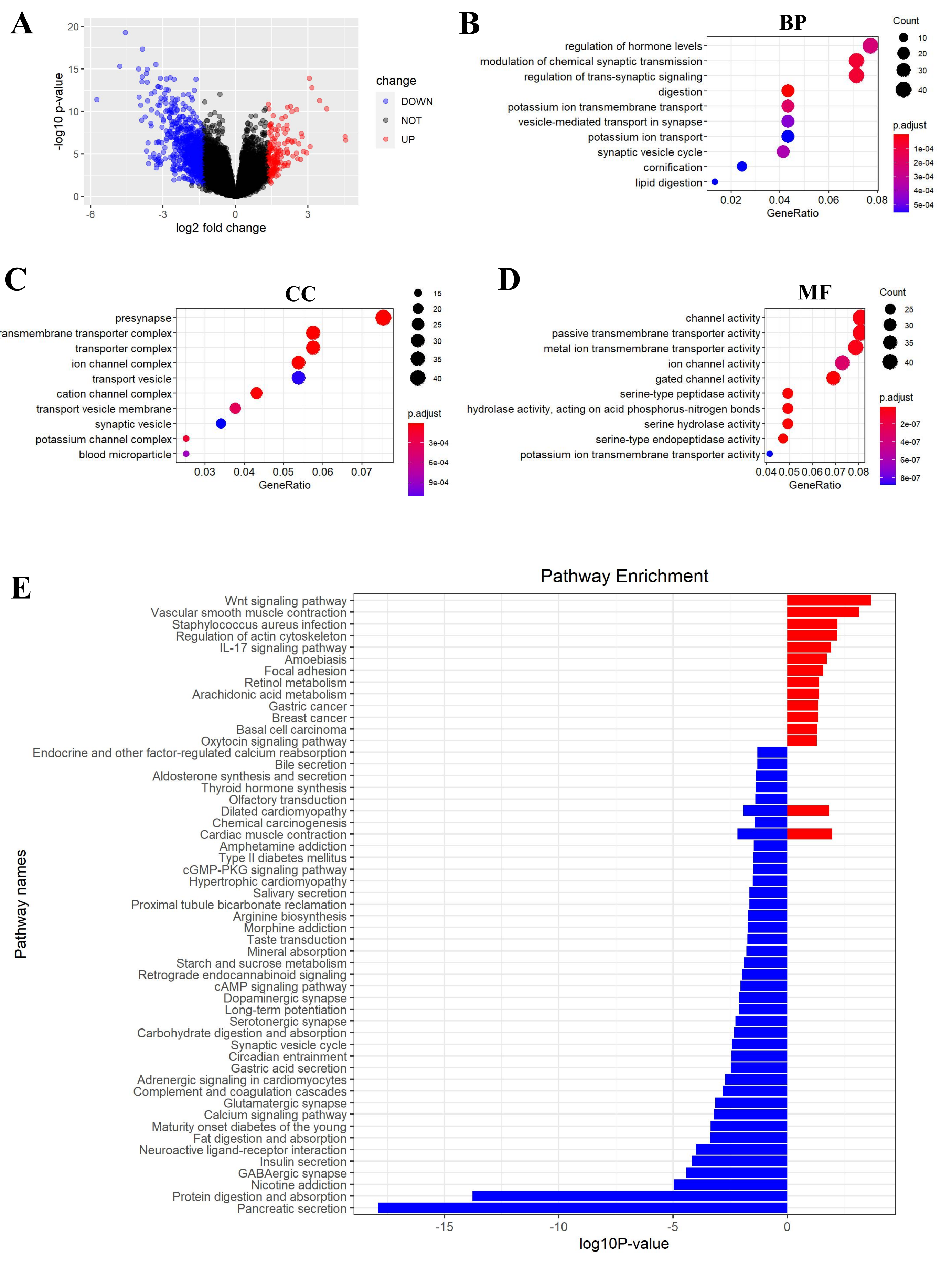
A
change
DOWN
NOT
UP
-log10 p-value
log2 fold change
B
BP
regulation of hormone levels
modulation of chemical synaptic transmission
regulation of trans-synaptic signaling
digestion
potassium ion transmembrane transport
vesicle-mediated transport in synapse
potassium ion transport
synaptic vesicle cycle
cornification
lipid digestion
GeneRatio
Count
p.adjust
C
CC
presynapse
transmembrane transporter complex
transporter complex
ion channel complex
transport vesicle
cation channel complex
transport vesicle membrane
synaptic vesicle
potassium channel complex
blood microparticle
GeneRatio
p.adjust
D
MF
channel activity
passive transmembrane transporter activity
metal ion transmembrane transporter activity
ion channel activity
gated channel activity
serine-type peptidase activity
hydrolase activity, acting on acid phosphorus-nitrogen bonds
serine hydrolase activity
serine-type endopeptidase activity
potassium ion transmembrane transporter activity
GeneRatio
Count
p.adjust
E
Pathway Enrichment
Wnt signaling pathway
Vascular smooth muscle contraction
Staphylococcus aureus infection
Regulation of actin cytoskeleton
IL-17 signaling pathway
Amoebiasis
Focal adhesion
Retinol metabolism
Arachidonic acid metabolism
Gastric cancer
Breast cancer
Basal cell carcinoma
Oxytocin signaling pathway
Endocrine and other factor-regulated calcium reabsorption
Bile secretion
Aldosterone synthesis and secretion
Thyroid hormone synthesis
Olfactory transduction
Dilated cardiomyopathy
Chemical carcinogenesis
Cardiac muscle contraction
Amphetamine addiction
Type II diabetes mellitus
cGMP-PKG signaling pathway
Hypertrophic cardiomyopathy
Salivary secretion
Proximal tubule bicarbonate reclamation
Arginine biosynthesis
Morphine addiction
Taste transduction
Mineral absorption
Starch and sucrose metabolism
Retrograde endocannabinoid signaling
cAMP signaling pathway
Dopaminergic synapse
Long-term potentiation
Serotonergic synapse
Carbohydrate digestion and absorption
Synaptic vesicle cycle
Circadian entrainment
Gastric acid secretion
Adrenergic signaling in cardiomyocytes
Complement and coagulation cascades
Glutamatergic synapse
Calcium signaling pathway
Maturity onset diabetes of the young
Fat digestion and absorption
Neuroactive ligand-receptor interaction
Insulin secretion
GABAergic synapse
Nicotine addiction
Protein digestion and absorption
Pancreatic secretion
Pathway names
log10P-value

Figure 7

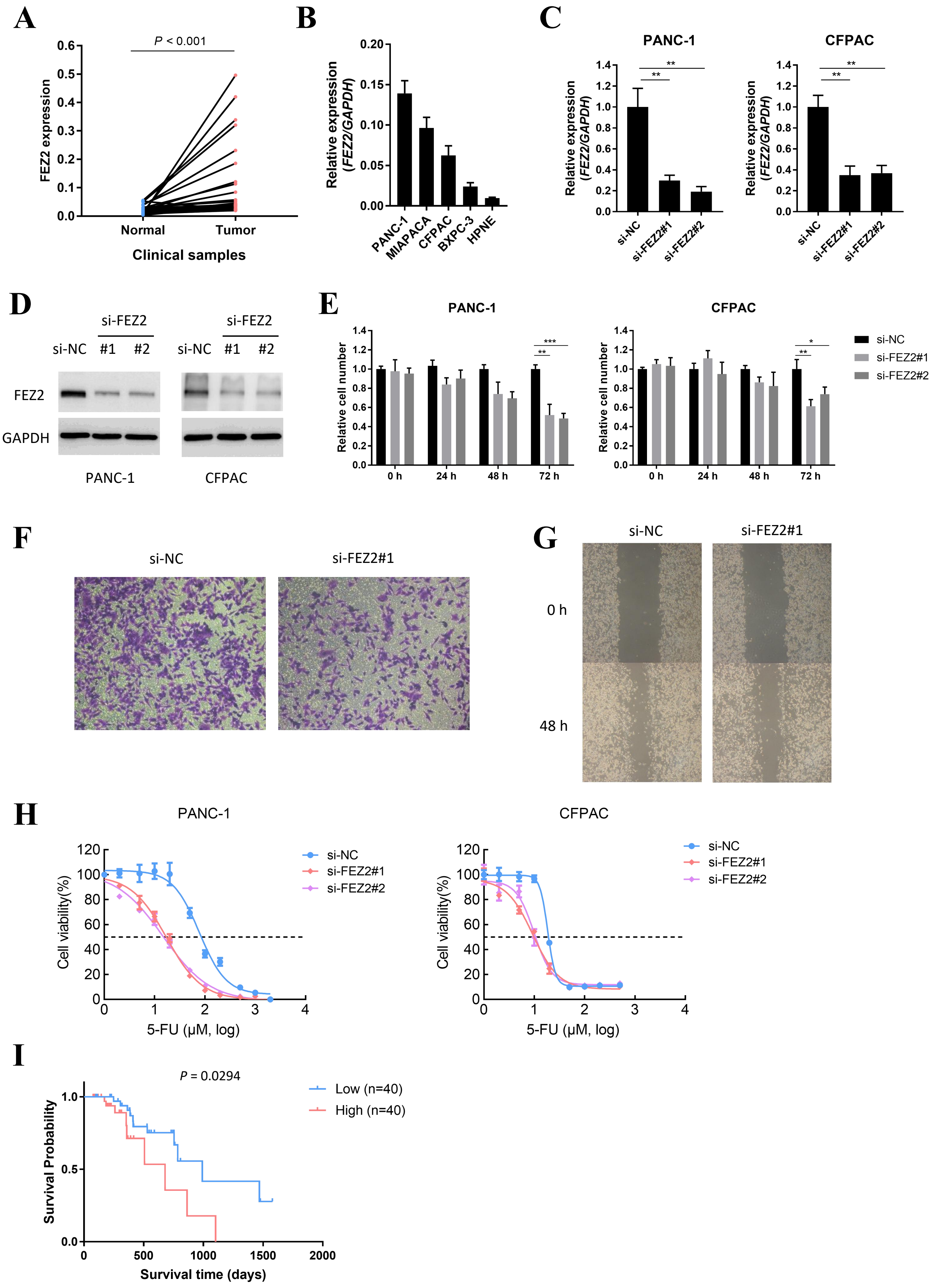
A
P < 0.001
FEZ2 expression
Normal
Tumor
Clinical samples
B
Relative expression (FEZ2/GAPDH)
PANC-1
MIAPACA
CFPAC
BXPC-3
HPNE
C
PANC-1
CFPAC
Relative expression (FEZ2/GAPDH)
si-NC
si-FEZ2#1
si-FEZ2#2
D
si-FEZ2
si-NC #1 #2
FEZ2
GAPDH
PANC-1
CFPAC
E
PANC-1
CFPAC
Relative cell number
0 h
24 h
48 h
72 h
si-NC
si-FEZ2#1
si-FEZ2#2
F
si-NC
si-FEZ2#1
G
si-NC
si-FEZ2#1
0 h
48 h
H
PANC-1
CFPAC
Cell viability(%)
5-FU (μM, log)
si-NC
si-FEZ2#1
si-FEZ2#2
I
P = 0.0294
Survival Probability
Survival time (days)
Low (n=40)
High (n=40)
