# Supplementary figures and images for: Identification of FEZ2 as a potential oncogene in pancreatic ductal adenocarcinoma
Source: PeerJ. 2022 Jan 5;10:e12736. doi: 10.7717/peerj.12736 (PMC8742541; doi:10.7717/peerj.12736)

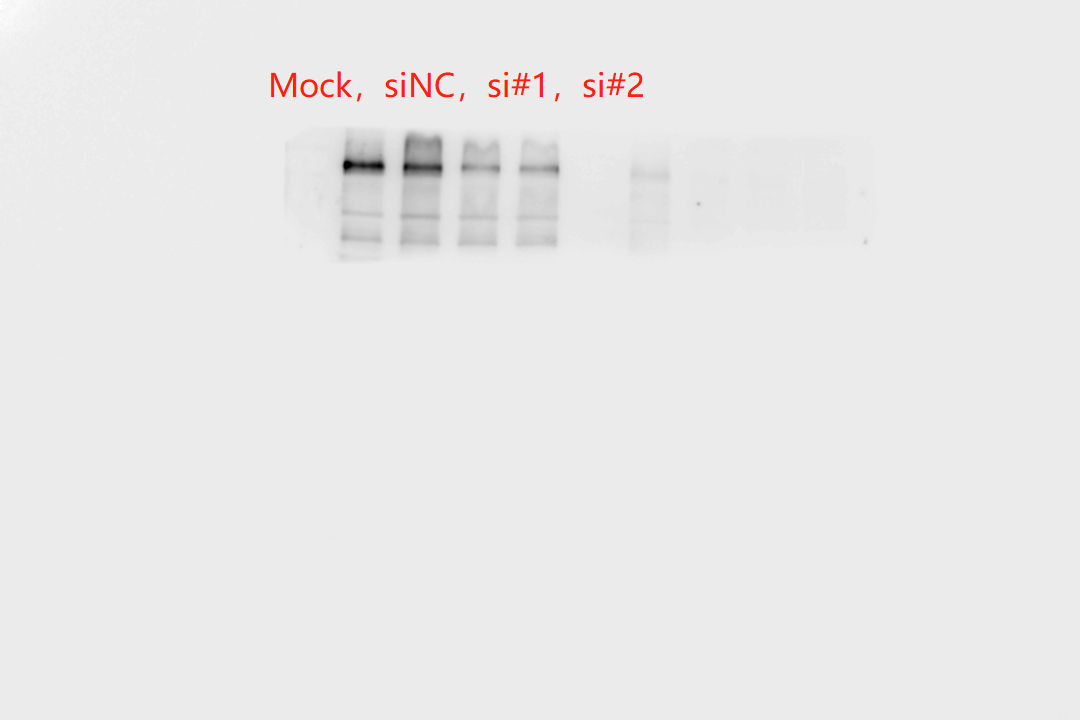

Supplement: Supplemental Information 4 [file peerj-10-12736-s004.zip › WB/cfpac fez2.png]

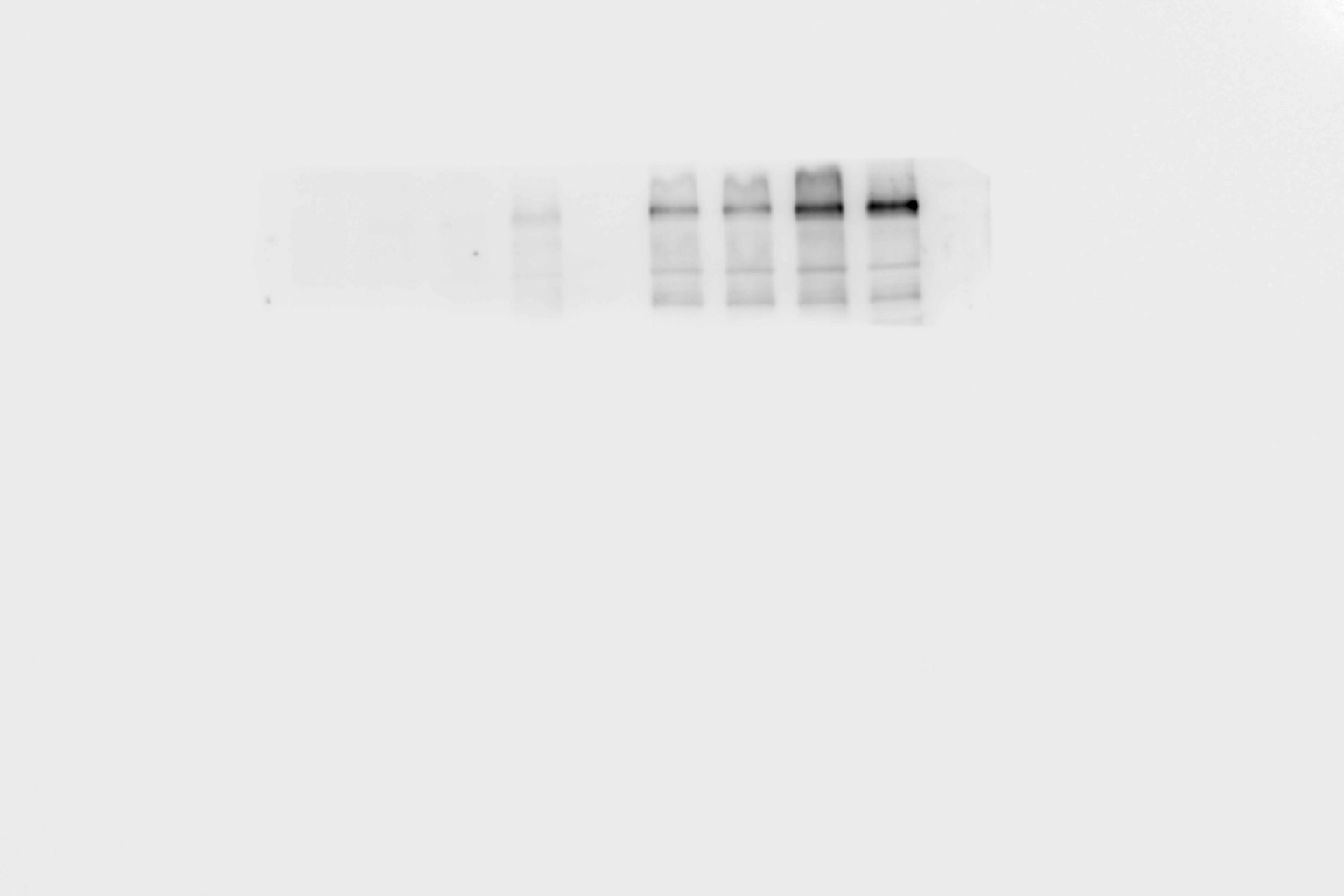

Supplement: Supplemental Information 4 [file peerj-10-12736-s004.zip › WB/FEZ2 cfpac.jpg]

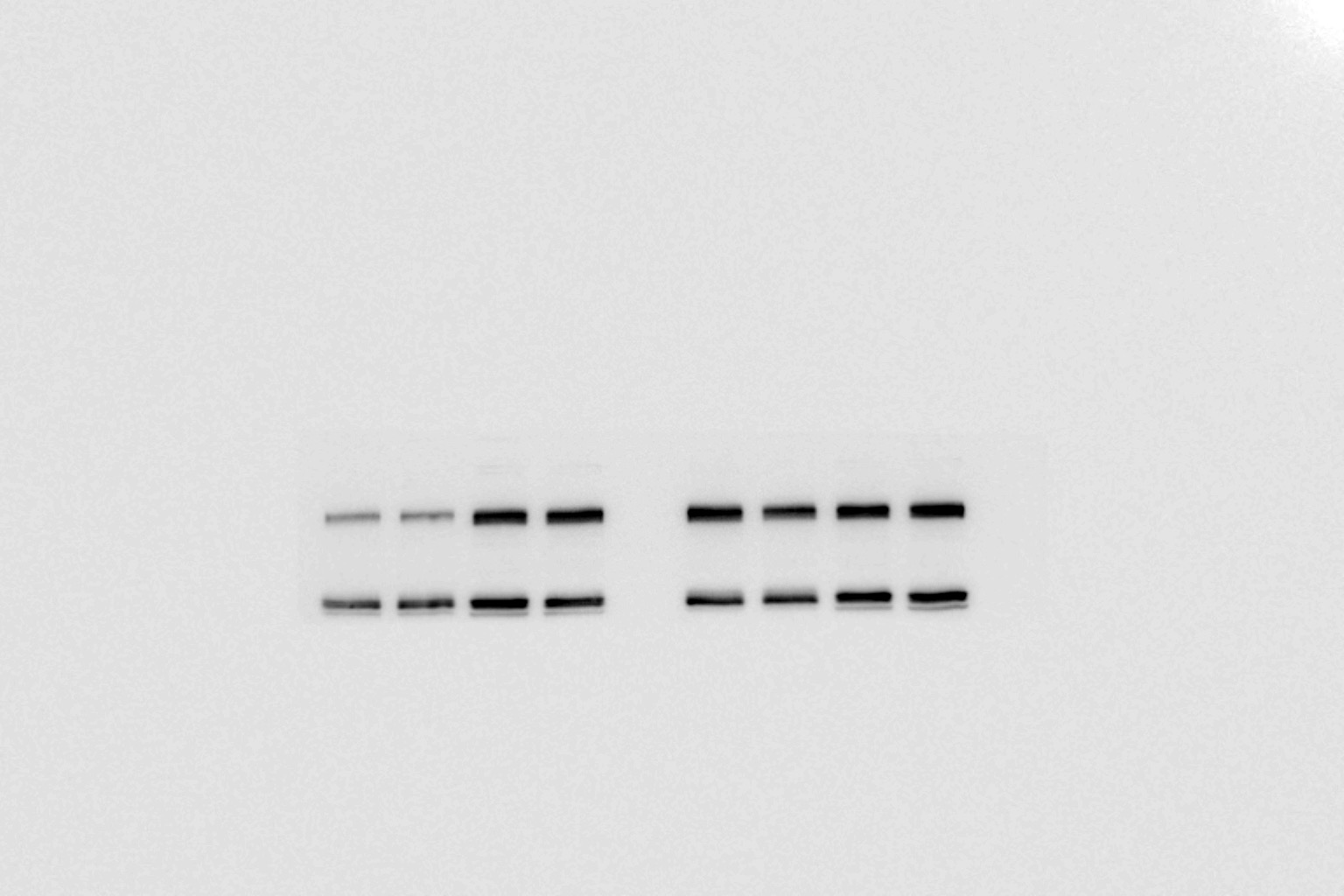

Supplement: Supplemental Information 4 [file peerj-10-12736-s004.zip › WB/FEZ2 panc1.jpg]

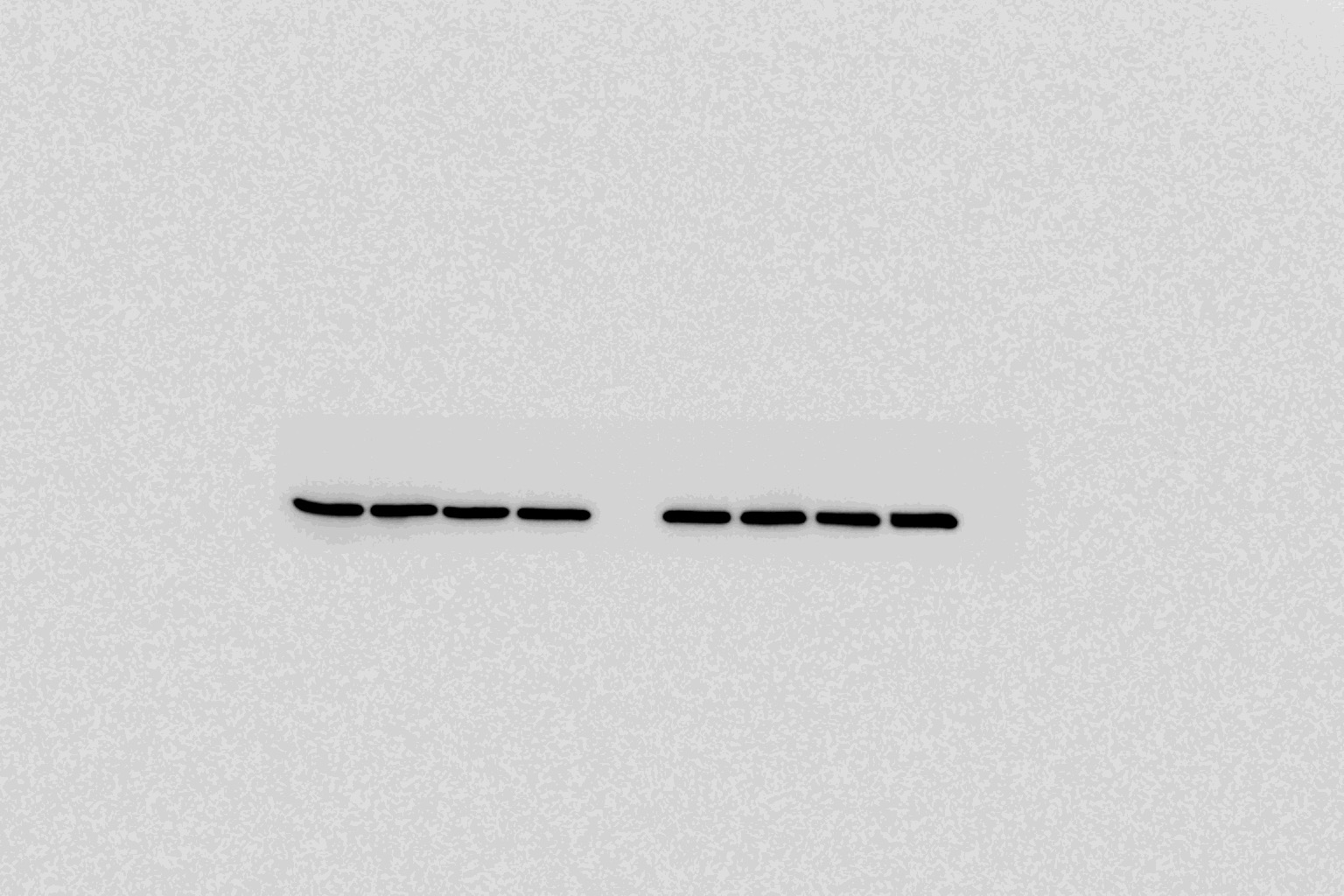

Supplement: Supplemental Information 4 [file peerj-10-12736-s004.zip › WB/gapdh1.jpg]

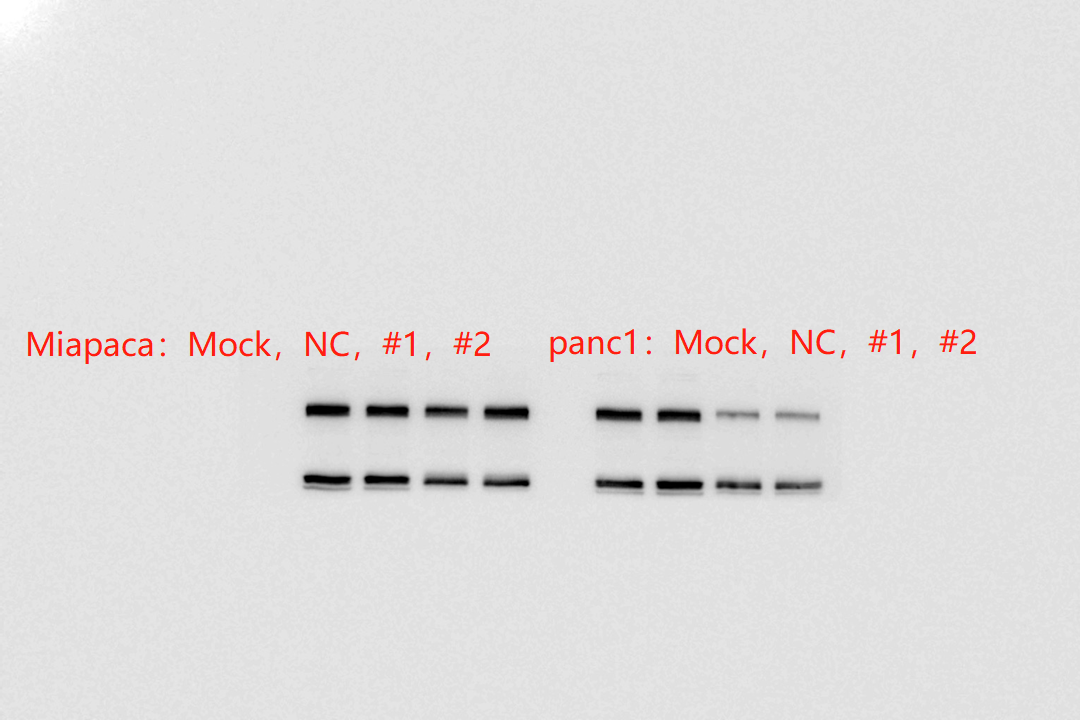

Supplement: Supplemental Information 4 [file peerj-10-12736-s004.zip › WB/panc1 fez2.png]

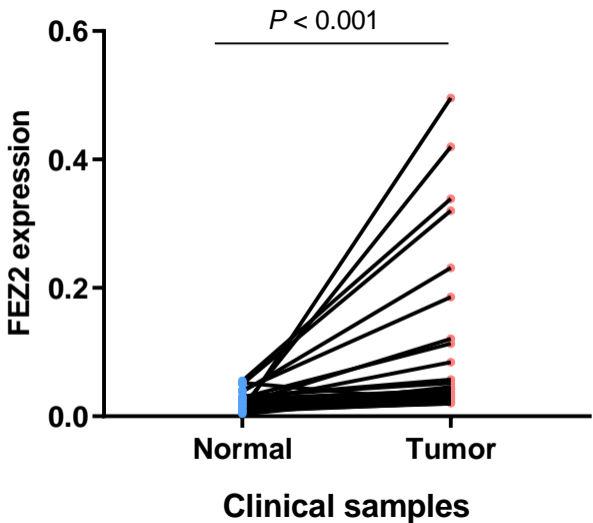

Supplement: Supplemental Information 8 — 50 pairs tissues [file peerj-10-12736-s008.pdf]

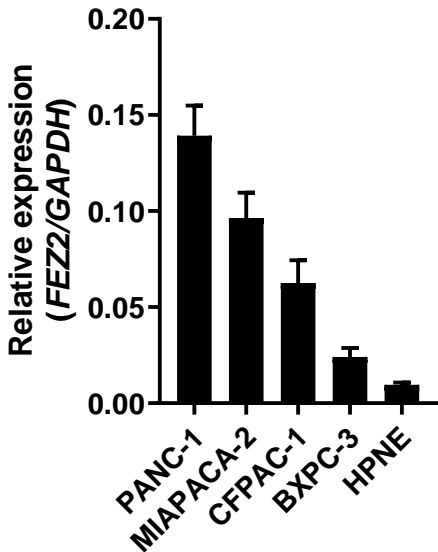

Supplement: Supplemental Information 9 [file peerj-10-12736-s009.pdf]

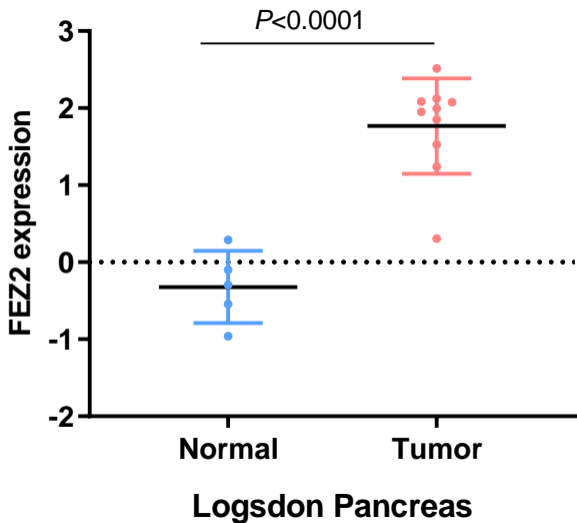

Supplement: Supplemental Information 10 [file peerj-10-12736-s010.pdf]

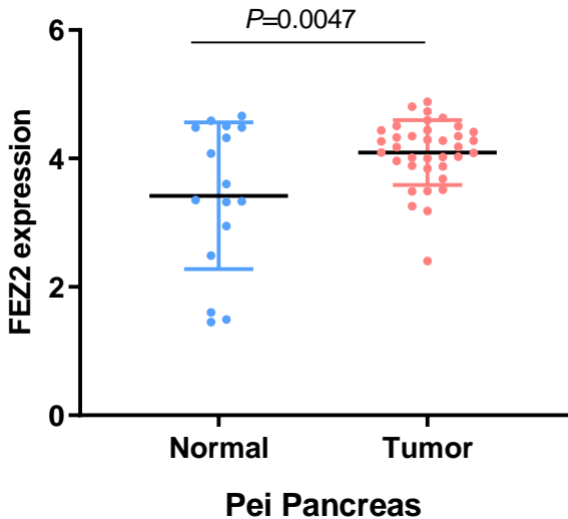

Supplement: Supplemental Information 11 [file peerj-10-12736-s011.pdf]

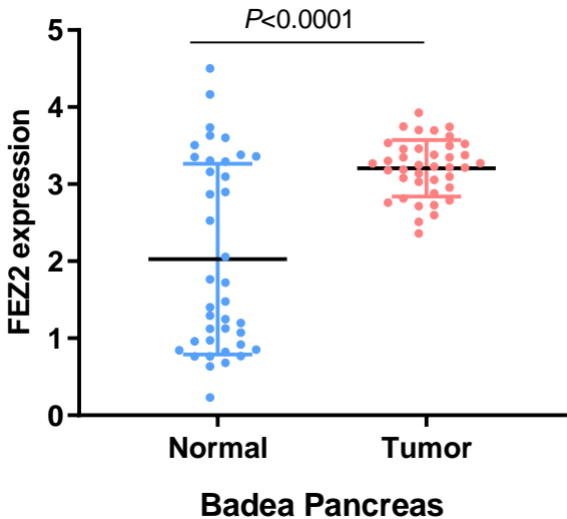

Supplement: Supplemental Information 12 [file peerj-10-12736-s012.pdf]

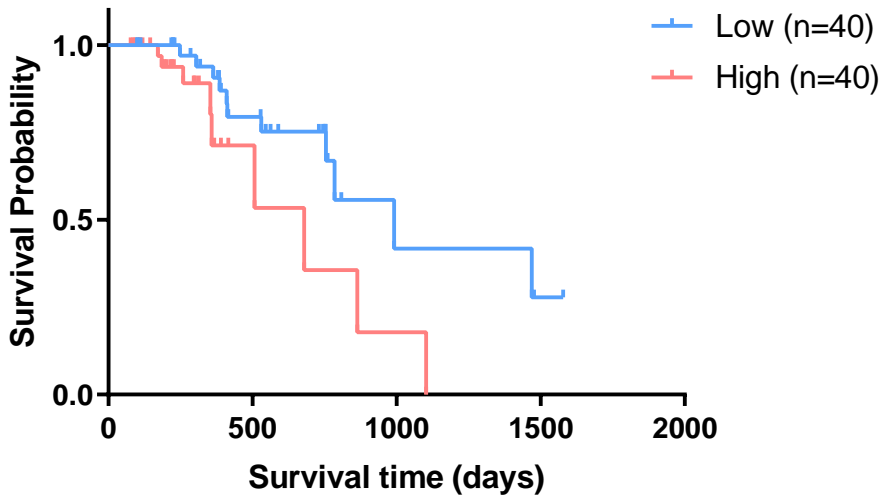

Supplement: Supplemental Information 13 [file peerj-10-12736-s013.pdf]

# CFPAC

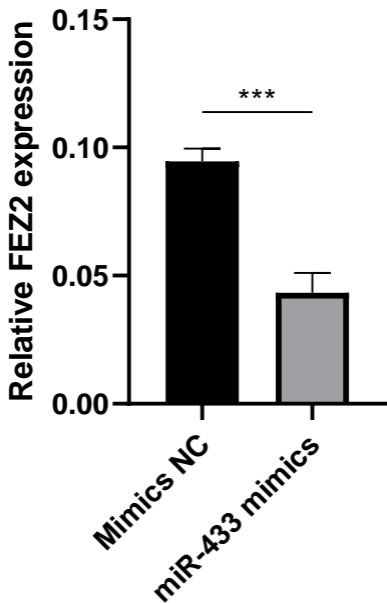

Supplement: Supplemental Information 14 [file peerj-10-12736-s014.pdf]

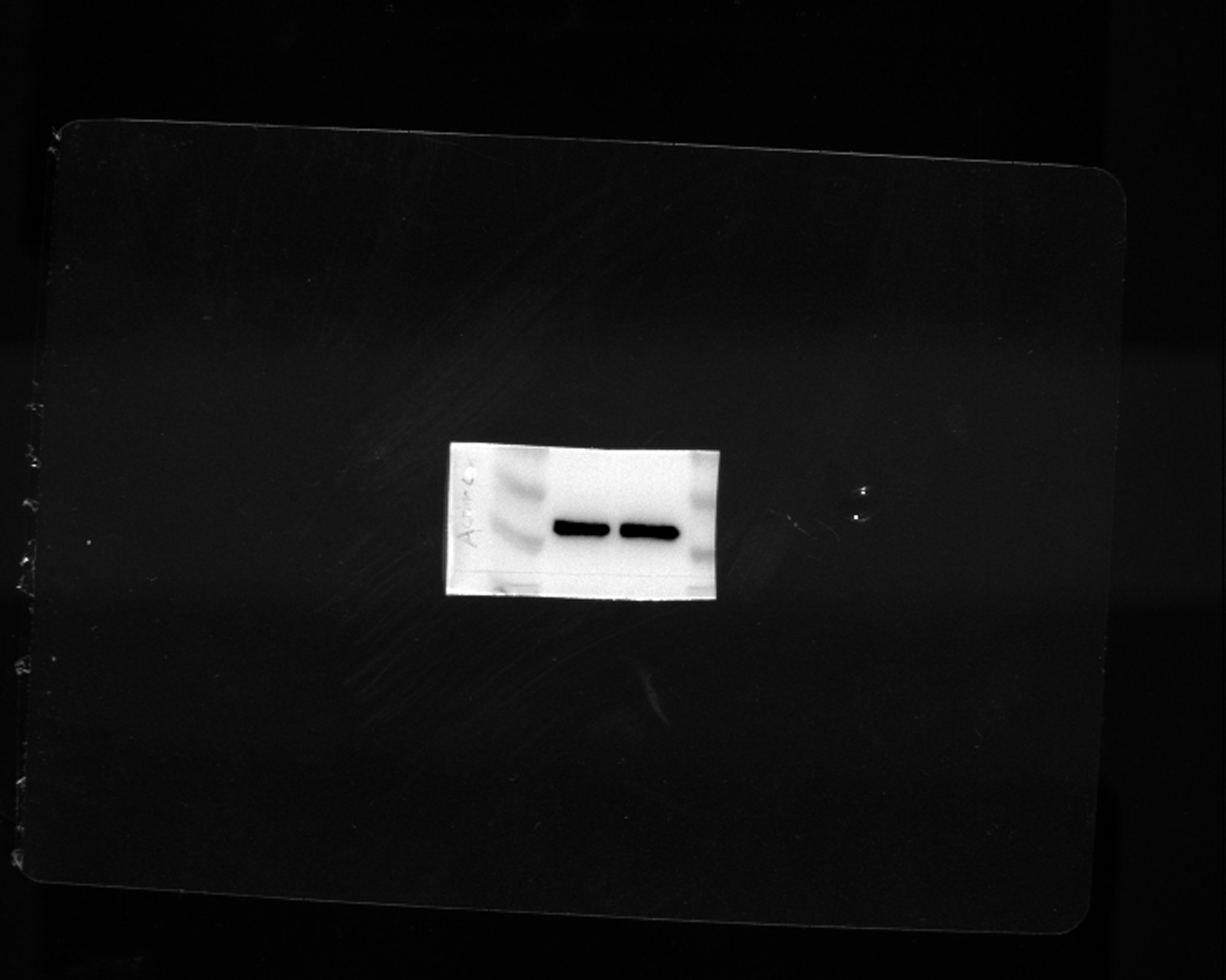

Supplement: Supplemental Information 15 [file peerj-10-12736-s015.zip › western-blot/actin_cf/actin_cf.tif]

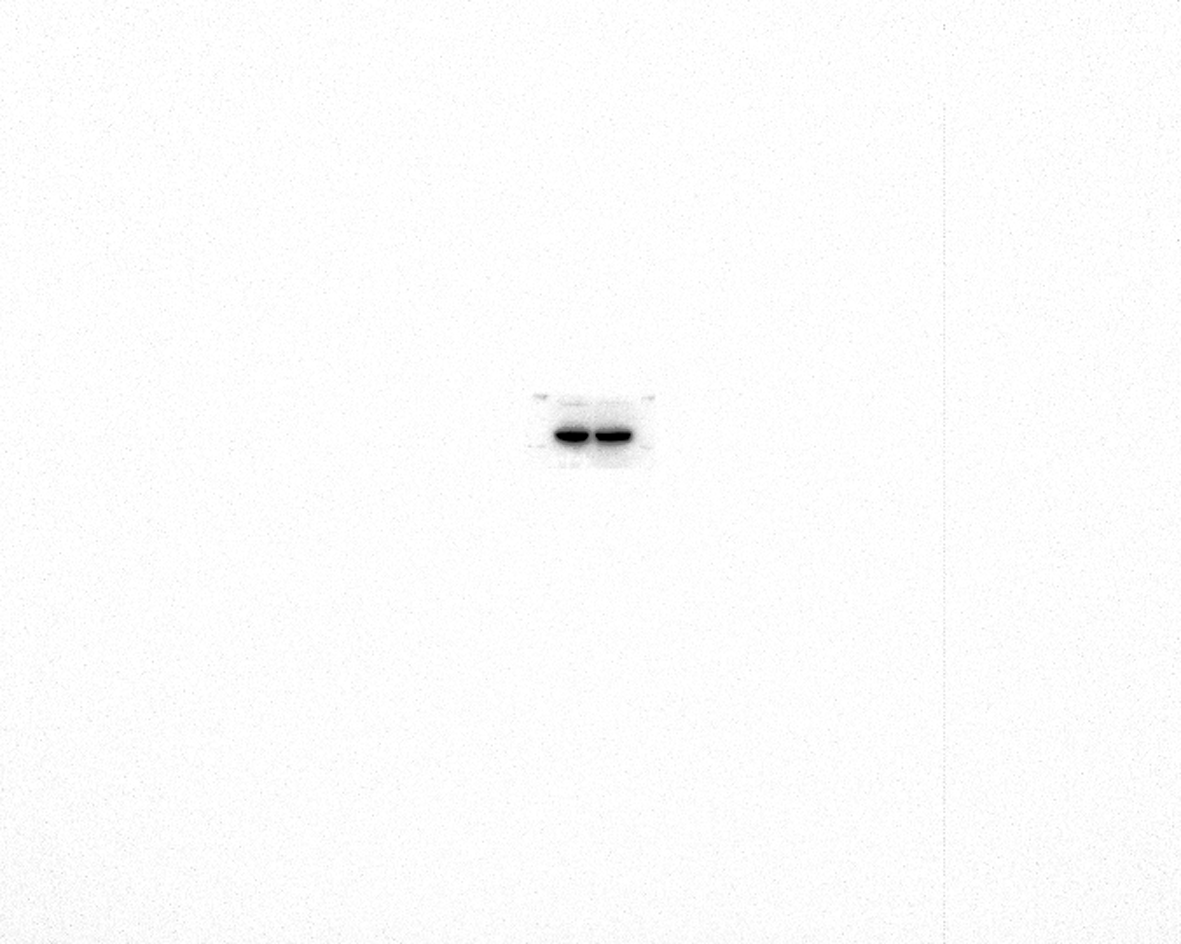

Supplement: Supplemental Information 15 [file peerj-10-12736-s015.zip › western-blot/actin_p_c/actin_p_c.tif]

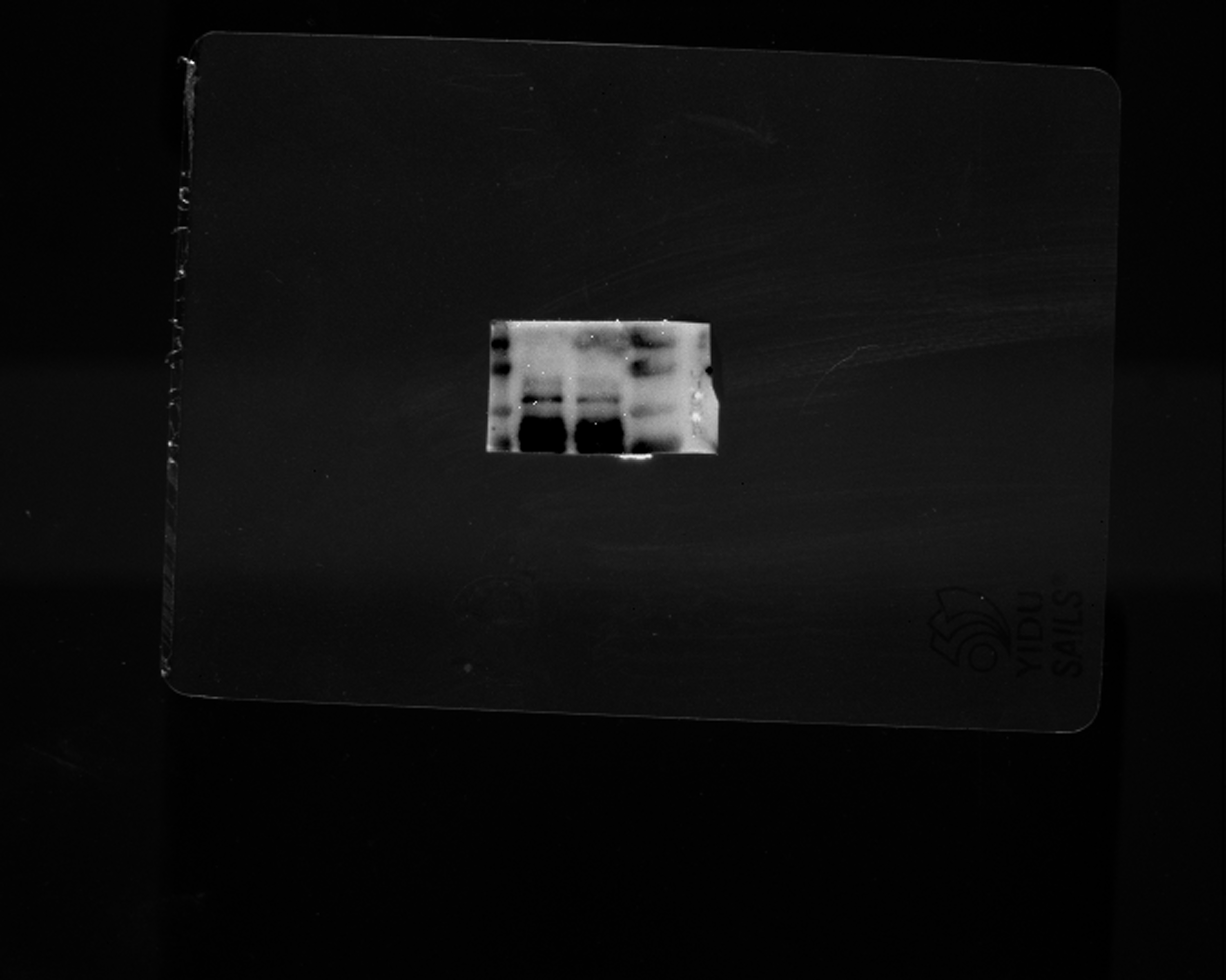

Supplement: Supplemental Information 15 [file peerj-10-12736-s015.zip › western-blot/cfpac_beta_catenin/cfpac_beta_catenin.tif]

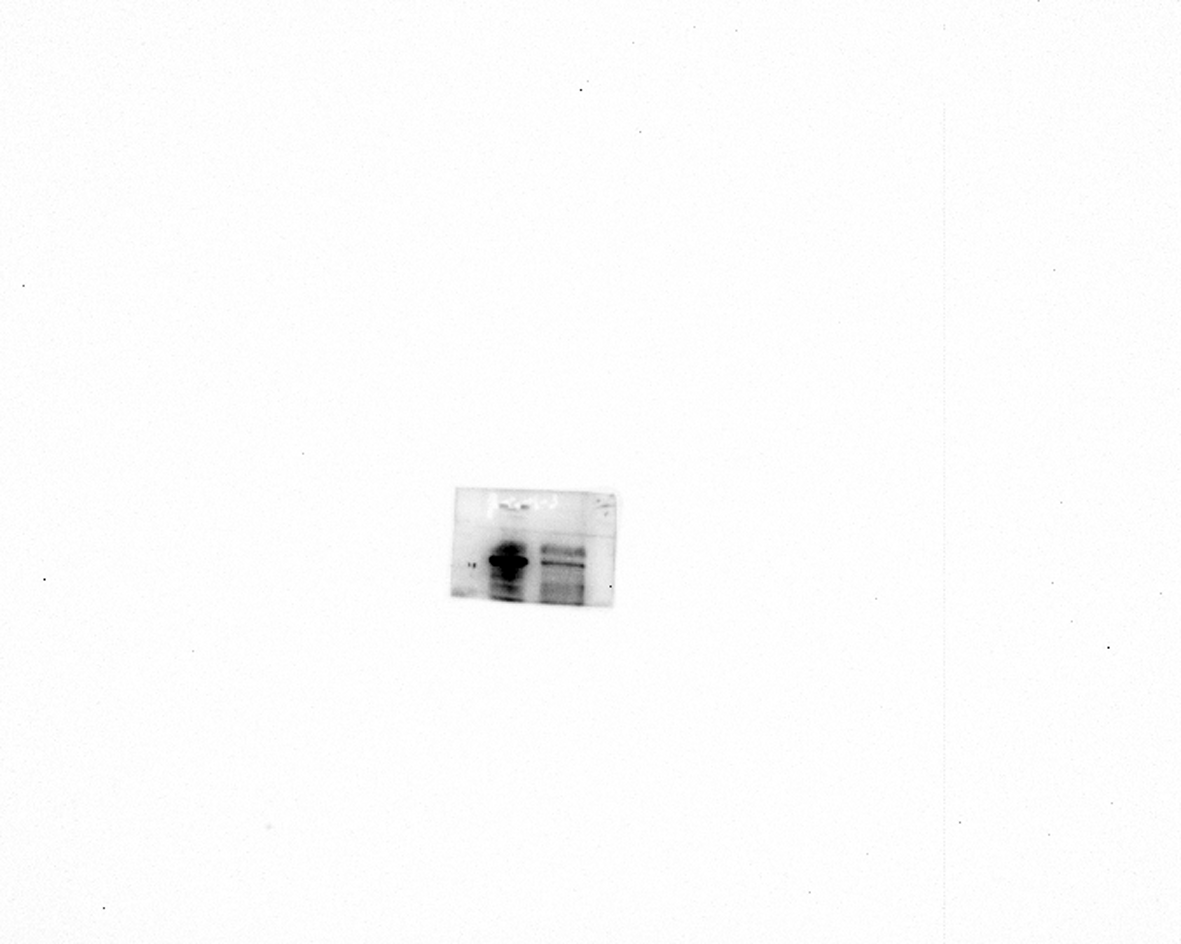

Supplement: Supplemental Information 15 [file peerj-10-12736-s015.zip › western-blot/p_b_cantenin_1_3/p_b_cantenin_1_3.tif]

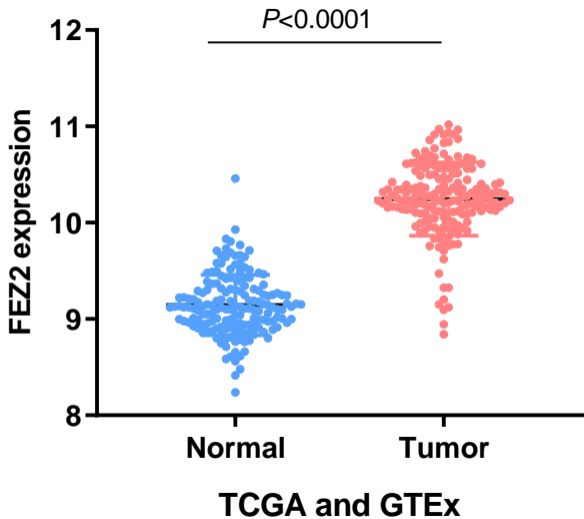

Supplement: Supplemental Information 16 [file peerj-10-12736-s016.pdf]

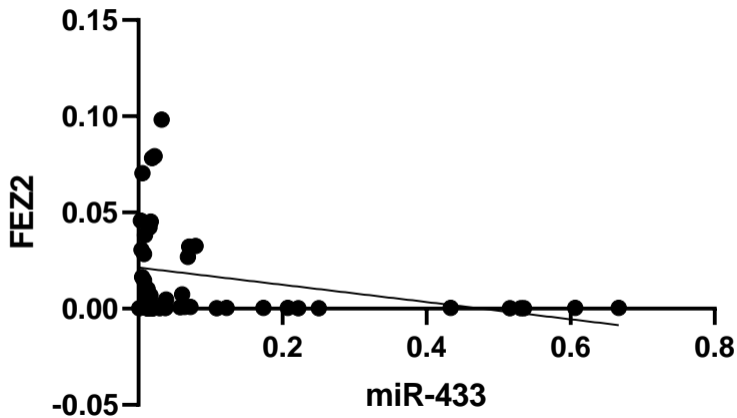

Supplement: Supplemental Information 17 [file peerj-10-12736-s017.pdf]

# CFPAC

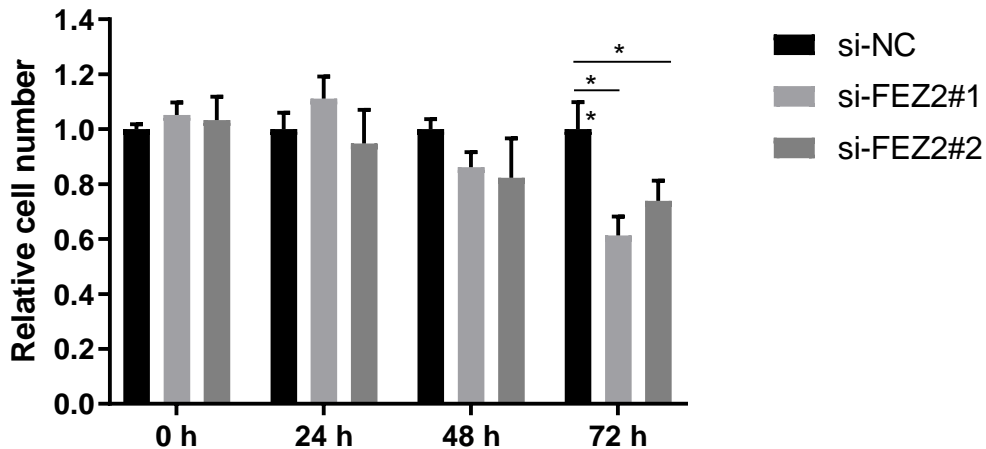

Supplement: Supplemental Information 18 — NC siFEZ2#1 siFEZ2#2 [file peerj-10-12736-s018.pdf]

# PANC-1

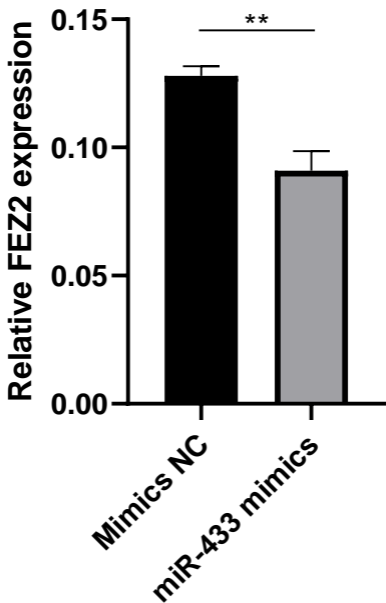

Supplement: Supplemental Information 19 [file peerj-10-12736-s019.pdf]

# CFPAC

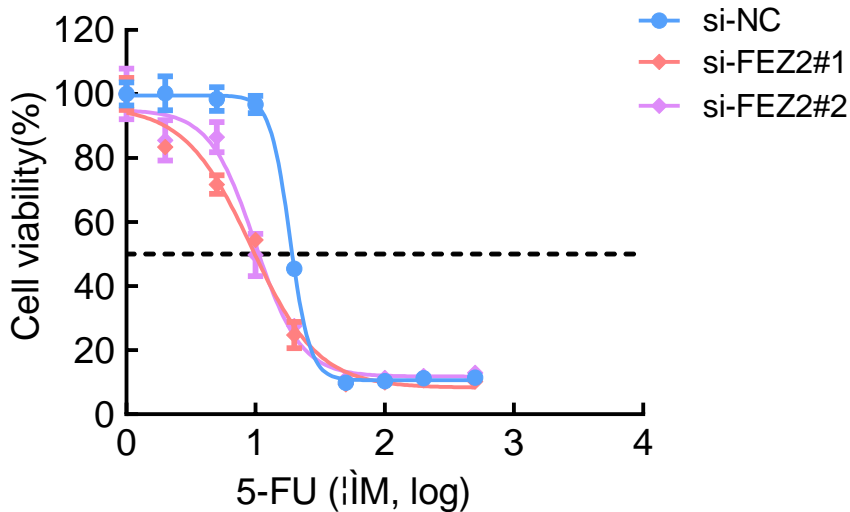

Supplement: Supplemental Information 20 — NC siFEZ2#1 siFEZ2#2 [file peerj-10-12736-s020.pdf]

# CFPAC

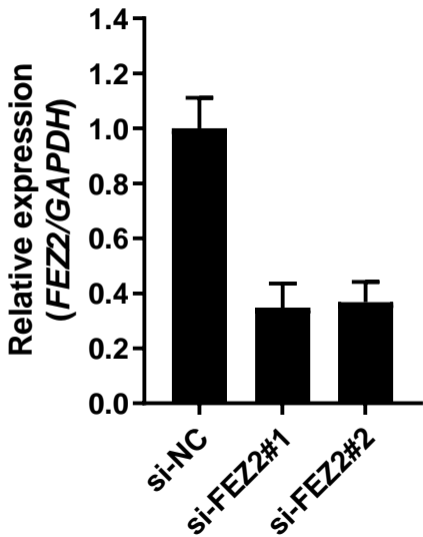

Supplement: Supplemental Information 21 — Cfpac-fez2 knock down [file peerj-10-12736-s021.pdf]

# PANC-1

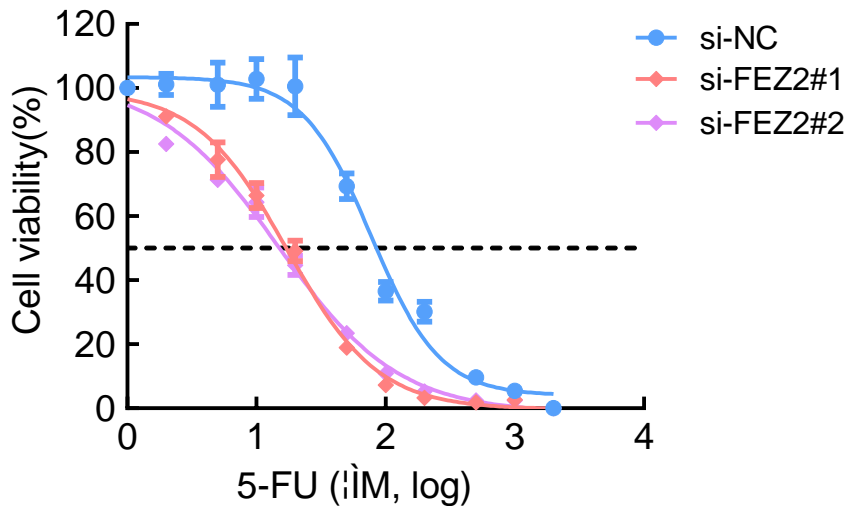

Supplement: Supplemental Information 22 [file peerj-10-12736-s022.pdf]

# PANC-1

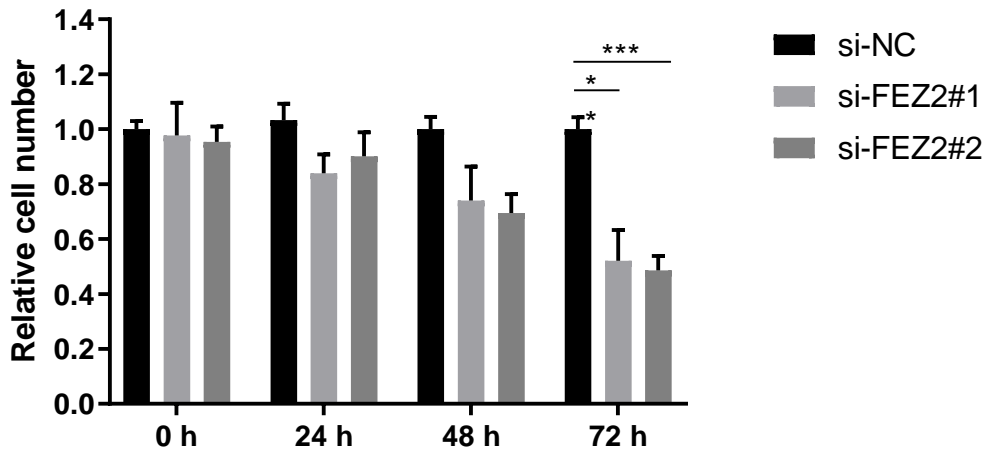

Supplement: Supplemental Information 23 — NC si-FEZ2#1 si-FEZ2#2 [file peerj-10-12736-s023.pdf]

# PANC-1

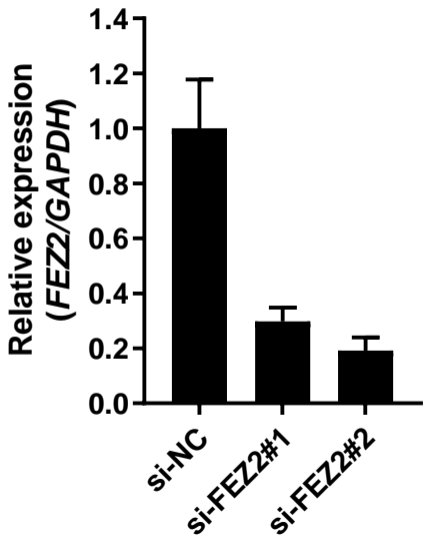

Supplement: Supplemental Information 24 — NC si-FEZ2#1 si-FEZ2#2 [file peerj-10-12736-s024.pdf]
